# KSHV inhibits stress granule formation by viral ORF57 blocking PKR activation

**DOI:** 10.1371/journal.ppat.1006677

**Published:** 2017-10-30

**Authors:** Nishi R. Sharma, Vladimir Majerciak, Michael J. Kruhlak, Zhi-Ming Zheng

**Affiliations:** 1 Tumor Virus RNA Biology Section, RNA Biology Laboratory, Center for Cancer Research, National Cancer Institute, NIH, Frederick, Maryland, United States of America; 2 Experimental Immunology Branch, Center for Cancer Research, National Cancer Institute, NIH, Bethesda, Maryland, United States of America; Florida State University, UNITED STATES

## Abstract

TIA-1 positive stress granules (SG) represent the storage sites of stalled mRNAs and are often associated with the cellular antiviral response. In this report, we provide evidence that Kaposi’s sarcoma-associated herpesvirus (KSHV) overcomes the host antiviral response by inhibition of SG formation via a viral lytic protein ORF57. By immunofluorescence analysis, we found that B lymphocytes with KSHV lytic infection are refractory to SG induction. KSHV ORF57, an essential post-transcriptional regulator of viral gene expression and the production of new viral progeny, inhibits SG formation induced experimentally by arsenite and poly I:C, but not by heat stress. KSHV ORF37 (vSOX) bearing intrinsic endoribonuclease activity also inhibits arsenite-induced SG formation, but KSHV RTA, vIRF-2, ORF45, ORF59 and LANA exert no such function. ORF57 binds both PKR-activating protein (PACT) and protein kinase R (PKR) through their RNA-binding motifs and prevents PACT-PKR interaction in the PKR pathway which inhibits KSHV production. Consistently, knocking down PKR expression significantly promotes KSHV virion production. ORF57 interacts with PKR to inhibit PKR binding dsRNA and its autophosphorylation, leading to inhibition of eIF2α phosphorylation and SG formation. Homologous protein HSV-1 ICP27, but not EBV EB2, resembles KSHV ORF57 in the ability to block the PKR/eIF2α/SG pathway. In addition, KSHV ORF57 inhibits poly I:C-induced TLR3 phosphorylation. Altogether, our data provide the first evidence that KSHV ORF57 plays a role in modulating PKR/eIF2α/SG axis and enhances virus production during virus lytic infection.

## Author summary

Mammalian RNA granules, including stress granules (SG), are important components of the host cell antiviral responses and their assembly is widely counteracted by RNA viruses. In Kaposi’s sarcoma-associated herpesvirus (KSHV) lytically infected B cells, we found that KSHV infection inhibits the assembly of SG by expression of viral lytic protein ORF57 and ORF37 (vSOX). KSHV ORF57 blocks arsenite-induced SG formation by binding to and preventing PACT from activating PKR. ORF57 also interacts with two double-stranded RNA binding motifs of PKR and prevents its binding with poly I:C and autophosphorylation, subsequent inhibition of eIF2α phosphorylation and SG formation. Consistently, knockdown of PKR increases production of KSHV virions. This function of KSHV ORF57 is conserved in homolog HSV-1 ICP27, but not in EBV EB2. We conclude that KSHV ORF57 antagonizes host antiviral defenses for virus lytic infection and production.

## Introduction

Mammalian somatic cells produce two types of RNA granules, processing bodies (P-bodies, PB) and stress granules (SG) [1,2]. Both granules are physically and mechanistically distinct compartments with many unique biomarkers. While GW182 is confined to PB, RNA-binding proteins TIA-1, poly(A) binding protein (PABP) and G3BP are specific markers of SG. PB appear during normal cell growth and contain enzymes for RNA de-capping and degradation [1,3], and have been shown to store and degrade siRNA- or miRNA-guided mRNA [4,5]. SG on the other hand, lack de-capping/de-adenylating machinery and appear during cell stress to play a role in global translational arrest by storing mRNA [1]. Therefore, SG represent a central and dynamic warehouse where stored mRNA is protected and exchanged with polysomes or PB for further translation or degradation, respectively [3,6].

SG contain 40S ribosomal subunits, mRNAs, dozens of RNA-binding proteins and many translation initiation factors including eIF4G, eIF4E, eIF3, and PABP [3,7,8]. SG assembly is initiated by phosphorylation of the α subunit in eIF2 at a specific serine (Ser 51) residue [9]. eIF2 is a translation initiation factor which forms a ternary complex with GTP and the initiator methionine-tRNA (eIF2-GTP-tRNA_i_-Met) [10] and in turn loads the initiator tRNA^met^ onto the small ribosomal subunit [11–13]). Different types of stress (oxidative, heat, or nutrient deprivation) can induce eIF2α phosphorylation by activation of four different eIF2α kinases (GCN2, PKR, PERK, and HRI) [14,15]. Phosphorylation of heterotrimeric eIF2 on its regulatory α subunit increases its affinity with eIF2β (the subunit responsible for GTP binding) and thus reduces its availability for GTP exchange. This deficiency in GTP exchange inhibits the ability of eIF2 to reach its active GTP-bound state and therefore prevents ternary complex formation and arrests translation initiation [16]. Consequently, polysomes disassemble leaving translationally arrested mRNPs to be recognized by the RNA binding proteins, TIA-1 and TIAR, and sequestered through their prion-like aggregation property to nucleate SG [17,18]. Many RNA binding proteins including tristetrapolin (TTP), and fragile X mental retardation proteins (FMRP) also join the assembly in SG [19,20].

Virus infection imposes stress on multiple biosynthetic pathways in host cells, including translation [21], and captures the host translation machinery to ensure virus translation and production [22]. During infection, the presence of viral double stranded RNA (dsRNA) activates host cell PKR to induce eIF2α phosphorylation and SG formation and thereby, triggers the host cell antiviral response and shutting down host cell translation [23,24]. To bypass this response, however, many RNA viruses utilize alternative ways of translation [25]. Notably, several RNA viruses suppress the formation of SG by a viral factor [26–30]. Poliovirus C3 cleaves Ras-GAP (Ras GTPase activating protein) SH3 domain-binding protein (G3BP), a component of SG that initiates the assembly of SG and interacts with inactive PKR [28,31,32]. Semliki Forest virus nsP3 targets G3BP [33], and influenza virus NS1 inactivates dsRNA-activated PKR [26,27]. Hepatitis C Virus instead induces SG formation, but co-opts SG proteins for its replication and production [34,35]. The regulation of SG formation during infection with large DNA viruses is poorly understood although herpesviruses are proposed to produce viral proteins to regulate SG formation [33,36,37].

Kaposi’s sarcoma-associated herpesvirus (KSHV) is a γ-2 herpesvirus [38] and infects human B lymphocytes and endothelial cells. KSHV infection leads to development of Kaposi’s sarcoma, primary effusion lymphoma (PEL), and multicentric Castleman’s disease (MCD) [39,40]. Like other herpesviruses, KSHV infection undergoes two alternative life-cycle programs. Viral lytic infection is characterized with the expression of all viral genes to produce infectious virions; whereas latent viral infection features highly restricted expression of only a few viral genes. Although the underlying mechanism responsible for the switch between lytic and latent infection, or vice versa, remains an unresolved topic, it is known that both viral and host factors are involved in the shift of KSHV infection [41,42]. A viral replication and transcription activator (RTA or ORF50) is an immediately early protein and is essential for transactivation of almost all other viral genes in the lytic infection. RTA expression from the latent KSHV infection can be induced experimentally by chemicals such as sodium n-butyrate (Bu) [43] or valproic acid (VA) [44,45]. Another important KSHV protein is ORF57 (mRNA transcript accumulation or MTA) which is an early lytic RNA-binding protein responsible for posttranscriptional processing of viral transcripts and virus production [46,47]. ORF57 stabilizes viral RNAs [48–51]), promotes splicing of intron-containing viral mRNA [52,53], and inhibits miRNA function to promote viral gene expression [48,54] through its interactions with targeted RNA and numerous host factors [47]. In this report, we discovered a novel function of ORF57 to inhibit SG formation during KSHV lytic infection. Mechanistically, ORF57 directly interacts with PACT and PKR and prevents phosphorylation of PKR and eIF2α and, thereby, prevents both SG formation and the stalling of RNA translation.

## Results

### KSHV-infected cells are refractory to SG induction during lytic infection

**Fig 1:**
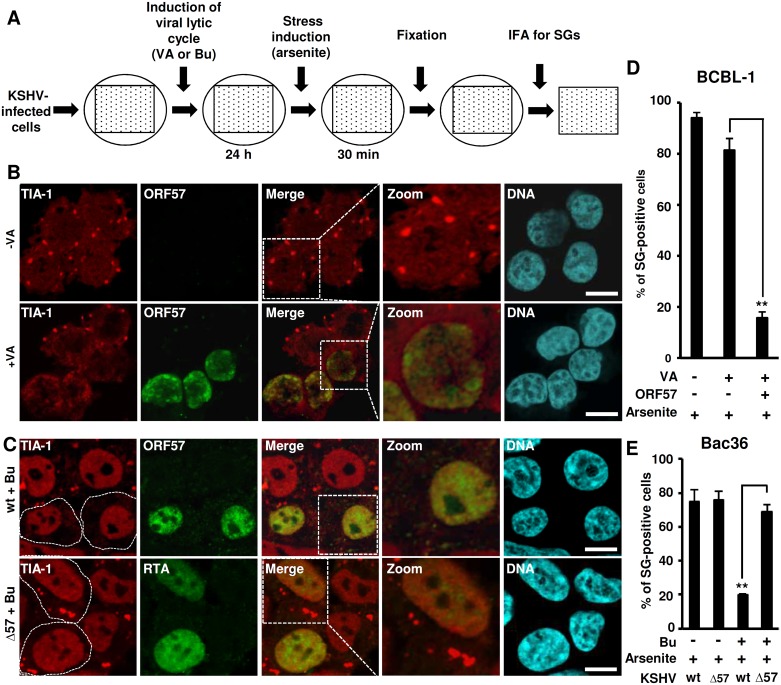
KSHV latent infection is permissive for SG formation while the viral lytic infection with ORF57 expression is refractory. (A) Outlines of KSHV reactivation and stress granule induction in BCBL-1 or Bac36 cells. Circles, culture dishes; dotted squares, cells grown on cover slips. (B and C) Induction of SG by arsenite in KSHV-infected BCBL-1 and Bac36 cells. KSHV lytic infection in BCBL-1 was induced by valproic acid (VA, 1 mM) (B) and in Bac36 cells harboring a wild-type or ORF57-null KSHV genome (wt or Δ57) was induced by sodium butyrate (Bu, 3 mM) (C). The cells with or without VA or Bu induction for 24 h were further treated with 0.5 mM arsenite for 30 min and then immunostained for the SG-specific marker TIA-1 and viral lytic protein ORF57 (BCBL-1 cells and Bac36 wt cells) or RTA (Bac36 Δ57 cells). Bac36 cells with ORF57 or RTA expression (C) are separated from the cells without ORF57 or RTA expression by dashed white borderlines. The nuclei were counterstained with Hoechst dye. Scale bar = 10 μm. (D and E) Proportion of BCBL-1 and Bac36 cells with SG formation before and after virus lytic reactivation. Total of 50 cells in each group, ORF57-positive or RTA-positive (for Δ57 Bac36 cells) cells vs ORF57-negative or RTA-negative (for Δ57 Bac36 cells) cells, were counted in each experiment. The error bars represent SD from three independent experiments. **P<0.01 in Chi-squared test.

To determine whether KSHV is able to modulate SG formation in infected cells, we employed a defined strategy (Fig 1A) to study SG formation in KSHV-infected BCBL-1 cells [55] and HEK293-derived Bac36 cells [45] by arsenite, a common chemical inducer which causes oxidative stress and robust formation of SG [10]. Both BCBL-1 and Bac36 cells harbor an episomal KSHV genome at the latent stage and can be reactivated to lytic KSHV infection in the presence of 1 mM Valproic acid (VA) or 3 mM Butyrate (Bu) (Fig 1A) [45,55,56].

On cells with or without virus lytic induction, we performed IF staining for SG-specific TIA-1, an RNA-binding protein that promotes the assembly of SG [18]. In the absence of arsenite treatment BCBL-1 cells either with latent or VA-induced lytic KSHV infection showed no visible SG (S1A Fig). In contrast, after arsenite treatment the majority of cells displayed ~3–6 TIA-1-positive SG per cell (Fig 1B). These arsenite-induced SG were also stained positive for PABPC1 and G3BP, two other SG-specific markers (S1B and S1C Fig). However, BCBL-1 cells expressing viral lytic genes, indicated by the presence of ORF57, exhibited a remarkable reduction of arsenite-induced SG (Fig 1B, S1B and S1C Fig), both by numbers of SG per cell and by numbers of cells with SG (Fig 1B and 1D bar graph). We also assessed the ability of Bac36 cells containing a wt KSHV genome (Bac36-wt) or ORF57-null KSHV genome (Bac36-Δ57) to prevent arsenite-induced SG formation during virus lytic infection, although viral lytic induction in Bac36 cells is less efficient than BCBL-1 cells [45]. We detected RTA in Bac36-Δ57 cells and ORF57 in Bac36-wt cells for butyrate-induced expression of viral lytic genes. While the ORF57 expressing Bac36-wt cells showed complete abrogation of arsenite-induced SG formation, the Bac36-Δ57 cells with RTA expression in lytic infection did not (Fig 1C and 1E bar graph), nor in LANA or ORF45-expressing cells (S1D Fig). The TIA-positive granules induced by arsenite in Bac36-Δ57 cells are *bona fide* SG and were sensitive to cycloheximide [6] by which blocks the flux of molecules between fully formed SG and polysomes (S1E Fig). These data indicate that, unlike latent infection, the presence of viral early protein ORF57, but not viral RTA, ORF45 or LANA during KSHV lytic infection is capable of preventing the arsenite-induced SG formation.

**Fig 2:**
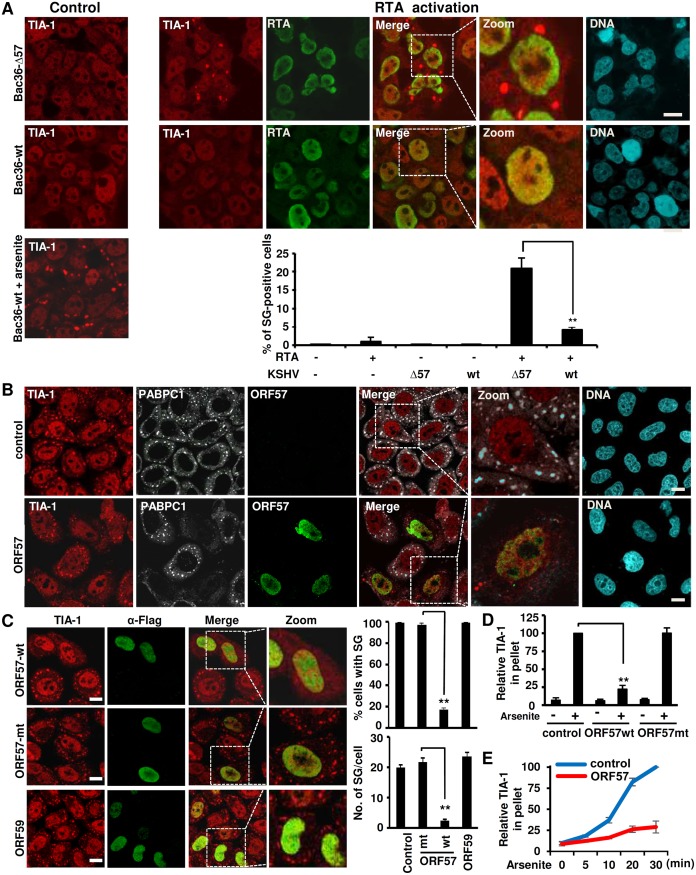
Viral ORF57 expression is required to suppress the formation of SG during KSHV lytic infection and under arsenite stress. (A) ORF57 expression is required to inhibit the formation of SG during KSHV lytic infection reactivated by RTA. Bac36 cells (wt or Δ57) were transfected either with a vector control (control) or with an RTA expression vector (RTA activation) to induce lytic KSHV infection. To observe SG, the cells were stained for TIA-1 (red) along with RTA (green) by each specific antibody. A positive control for SG was also set by arsenite treatment on Bac36 wt cells transfected with an empty vector. The nuclei were counterstained with Hoechst stain. Scale bar = 10 μm. Bar graph below the images shows the cells forming SG before and after virus lytic reactivation through RTA expression. HEK293 cells transfected with an empty or RTA expressing vector served as a negative control. Total of 100 cells in each group were counted in each experiment. The error bars represent mean ± SD (n = 3). **P<0.01 in Chi-squared test. (B) ORF57 alone is sufficient to inhibit SG formation. HeLa cells transfected with an ORF57-Flag (pVM7) expressing vector or an empty vector (control) for 24 h were induced with 0.5 mM arsenite for 30 min for SG formation. The cells were stained for ORF57 (green), SG-specific TIA-1 (red) and PABPC1 (white) by each corresponding antibody. The nuclei were counterstained with Hoechst stain. Scale bar = 10 μm. (C) The ORF57 N-terminal NLS is required to inhibit SG formation. HeLa cells were transfected either with an empty vector or a vector expressing ORF57-Flag (ORF57 wt), ORF57 mtNLS2+3-Flag (ORF57 mt) or ORF59-Flag (a viral DNA replication processivity factor) for 24 h and then induced by arsenite for 30 min for SG formation. Mouse anti-Flag antibody served to detect ORF57 wt, ORF57 mt and ORF59. TIA-1 antibody was used to probe SG. Scale bar = 10 μm. Bar graphs on the right are number of the cells with SG (upper) or number of SG per cell (lower) from at least 100 cells in each group. The error bar indicates mean± SD. **P<0.01 in Chi-squared test. (D) Relative amount of TIA-1 in insoluble pellets derived from HeLa cells with or without expression of functional ORF57. HeLa cells transfected with an ORF57 wt or ORF57 mt expressing vector or an empty Flag control vector for 24 h were either untreated (-) or treated (+) with 0.5 mM arsenite for 30 min to induce SG and then lysed in a sample buffer. The lysed samples were centrifuged at 15800 x g to separate soluble from insoluble fractions. The insoluble pellets containing SG were dissolved in SDS sample buffer for TIA-1 immunoblotting (S3B Fig). The relative amount of detectable TIA-1 in each sample, after normalized to tubulin loading control, was calculated over the amount of TIA-1 in arsenite-treated, Flag control pellets. **P<0.01 in student *t*-test. (E) Kinetic profile of TIA-1 in the insoluble pellets in the presence of an ORF57-expressing vector or an empty vector over the indicated time of arsenite treatment. Similar to Fig 2D, the insoluble pellets containing SG in HeLa cells with ORF57 (ORF57) or with an empty vector (control) were immunoblotted for the relative amount of TIA-1 in each time point (S3C Fig) and calculated as described above.

We also stained Bac36 cells (wt or Δ57) for TIA-1-positive SG in the absence of arsenite treatment, but in the presence of ectopic RTA expression to induce viral lytic infection. We did not see any visible SG in Bac36 wt or Δ57 cells (Fig 2A, control), unless the cells were treated with arsenite (Fig 2A, control). However, when cells are induced to the lytic phase by ectopic RTA expression, SG were found in approximate 20% of the RTA-expressing Bac36-Δ57 cells, but only in ~3% of the RTA-expressing Bac36-wt cells (Fig 2A for RTA activation Image and bar graph). Again, the RTA-induced SG formation in Bac36-Δ57 cells was sensitive to cyclohemixide treatment (S1F Fig) [6]. Importantly, the presence of ectopic RTA alone does not induce SG in KSHV-negative HEK293 cells, from where Bac36 cells were originated [45]. Collectively, these results indicate that KSHV lytic infection provides a stress to the infected cells and the expression of ORF57 but not RTA during virus lytic infection is required for suppression of SG formation.

### ORF57 expression is sufficient for inhibition of SG formation

The complex nature of lytic replication in KSHV makes it difficult to confirm that the observed suppression of SG formation is a result of ORF57 function. To examine whether ORF57 alone is sufficient to block SG formation in the absence of other viral lytic proteins, we transfected HeLa cells with an ORF57-expressing or an empty vector. Transfection of these plasmids did not induce SG formation as confirmed by staining for TIA-1 and PABPC1 (S2A Fig). We then treated the cells with arsenite for 30 min and performed similar immunostaining. As expected, all cells without ORF57 displayed SG positive for TIA-1, PABPC1 and G3BP1 staining, but a dramatic reduction in SG formation was found in ORF57-expressing cells (Fig 2B, S2B Fig), indicating that ORF57 is a viral lytic protein responsible for efficient suppression of SG formation.

There are three nuclear localization signals (NLS) present in the N-terminal domain of ORF57 that are important for the various functions of the viral protein. Introduction of point mutations in any two of the NLS renders ORF57 dysfunctional and unable to bind other partner proteins [57]. We next compared the ability of wild-type ORF57 (ORF57 wt) and an NLS mutant ORF57 (ORF57 mt) protein to abrogate arsenite-induced SG formation in HeLa cells. KSHV ORF59, a DNA polymerase processivity factor, served as another viral lytic protein control. While ORF57 wt showed nearly complete abrogation of SG formation, ORF57 mt and ORF59 did not (Fig 2C, panels on the left). By quantification, we measured ~20 SG per cell in all cells transfected with an empty vector, ORF57 mt, or ORF59, but ~80% of the ORF57-expressing cells did not exhibit any SG and the remaining ~20% of the cells showed a dramatically reduced number of SG (2–5 SG/cell) (Fig 2C, bar graphs on the right). Three-dimensional image reconstructions acquired by confocal microscopy verified the absence of SG in ORF57 expressing cells (S1 & S2 Videos). As an initial investigation into how ORF57 may prevent SG formation, we checked whether ORF57 affects the expression of TIA-1, PABPC1, G3BP, eIF4E and its phosphorylated form (p-eIF4E) and found no difference in the protein levels for all of these SG components, compared to control cells (S2C–S2E Fig).

During stress, TIA-1 aggregates in a similar manner to prion-like proteins to form SG [18]. The concentrated TIA-1 in SG can be detected in the insoluble pellets after high speed centrifugation of cellular extracts (S3A Fig). As expected, arsenite treatment of HeLa cells led to remarkable increase of TIA-1 in the pellet (Fig 2D and S3B Fig) in a time-dependent manner (Fig 2E and S3C Fig). Of particular interest, ORF57 wt, but not ORF57 mt, could be found in the pellets (S3B Fig) and prevent TIA-1 recruitment into the pellets (Fig 2D and S3C Fig). We calculated that ORF57 wt blocked ~75% of total TIA-1 from being aggregated in 30 min of arsenite treatment (Fig 2E) and this function of ORF57 begins even at 10 min of arsenite treatment of HeLa cells in this study (Fig 2E and S3C Fig). All together, these results indicate that a novel function of ORF57 is to establish the conditions that maintain the solubility of TIA-1 to prevent SG formation during stress.

### ORF57 inhibits the formation of SG by blocking eIF2α phosphorylation

**Fig 3:**
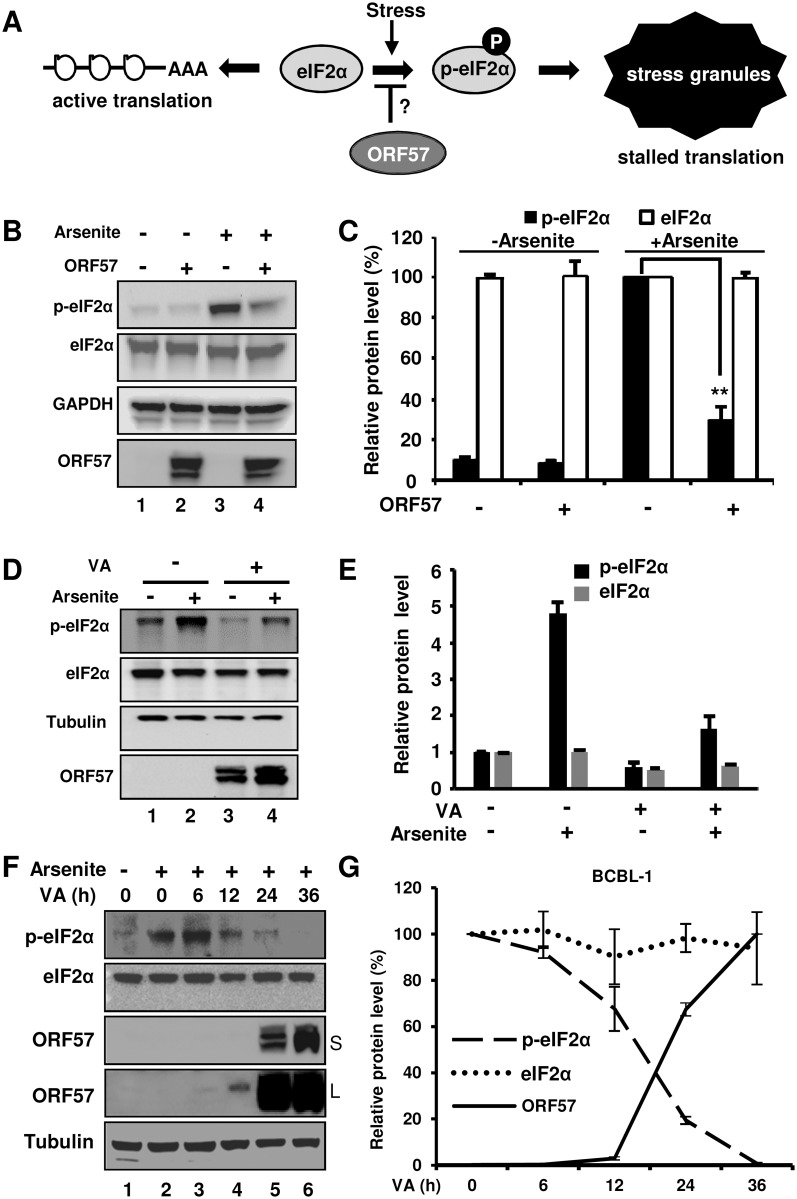
Viral ORF57 inhibits eIF2α phosphorylation. (A) Schematic diagrams showing the governing process of SG formation and the possible mechanism by which ORF57 may prevent it. (B) ORF57 inhibits eIF2α phosphorylation. HeLa cells transfected with an empty (-) or ORF57 expressing (ORF57) vector were treated with arsenite. The phosphorylated eIF2α (p-eIF2α) was measured by Western blot analysis using a Ser 51 phosphor-specific eIF2α antibody. Total level of eIF2α was determined by a pan-eIF2α antibody. GAPDH served as a loading control. (C) Graphical representation of the relative amount of p-eIF2α (black) and eIF2α (white) in the panel B. The relative intensity of the protein band in each sample, after normalizing to GAPDH, was calculated over that of the arsenite-treated empty vector control. The error bar indicates mean ± SD (n = 3). (D-E) KSHV lytic infection in BCBL-1 cells does not increase eIF2α phosphorylation, but rather decrease arsenite-induced eIF2α phosphorylation. BCBL-1 cells with or without VA induction for lytic virus infection was treated with or without arsenite for 30 min before cell lysate preparation for Western blotting with corresponding antibodies (D). ORF57 was blotted as an indication for viral lytic induction. Relative amount of total eIF2α or p-eIF2α in each sample after normalizing to tubulin was measured and plotted in bar graphs for comparison (E), with each protein level in lane 1 (D) being set to 1. (F and G) Kinetic ORF57 production and p-eIF2α reduction in BCBL-1 cells with KHSV lytic infection. BCBL-1 cells induced with 1 mM VA for the indicated time for virus lytic infection were treated with arsenite for 30 min and then analyzed by Western blotting (F). The un-induced cells without arsenite treatment served as a negative control. S, short time exposure; L, longer time exposure. The relative amount of each protein in each sample after normalizing to tubulin was plotted over the time when the sample was collected (G), with the protein level in arsenite-treated cells without VA induction (0) being set to 100%. The error bar indicates mean ± SD (n = 2).

The formation of SG is initiated as a downstream event after elF2α phosphorylation which leads to the prion-like aggregation of TIA-1. Normally, eIF2α is required to initiate mRNA translation by promoting the binding of tRNA^met^ to the 40S ribosome in a GTP-dependent manner. Stress induces phosphorylation of eIF2α to attenuate eIF2α activity (Fig 3A) and thereby promote TIA-1 aggregation to form the SG where mRNA translation is stalled [14].

Accordingly, we examined whether ORF57 could affect eIF2α phosphorylation. HeLa cells transfected with an ORF57-expressing or empty vector and treated with arsenite were blotted for the phosphorylation status of eIF2α (p-eIF2α). As expected, arsenite was found to induce eIF2α phosphorylation ~15 times greater than basal level (Fig 3B, lane 3 vs lane 1), whereas ORF57-expressing cells remarkably inhibited eIF2α phosphorylation upon arsenite induction (Fig 3B, lane 4 vs lane 3, and Fig 3C), with the total level of eIF2α protein remaining the same.

To correlate kinetic production of KSHV ORF57 with both total eIF2α and phosphorylated eIF2α, we induced KSHV-infected BCBL-1 cells with 1 mM VA for the indicated time and then treated the cells with arsenite for 30 min before collecting cell lysates for Western blotting analysis. Although lytic KSHV infection did not increase eIF2α phosphorylation over that of latent KSHV infection in the cells without exogenous stress (Fig 3D and 3E), we found that arsenite-induced phosphorylation of eIF2α was in reverse correlation with kinetic ORF57 expression (Fig 3F and 3G). Higher ORF57 expression resulted in less eIF2α phosphorylation, while the total eIF2α remained unchanged (Fig 3F and 3G). Altogether, these data indicate that ORF57 is inhibitory for eIF2α phosphorylation both when expressed alone and when present with other viral proteins during viral lytic infection.

### ORF57 disturbs the PKR pathway to inhibit eIF2α phosphorylation and SG formation

**Fig 4:**
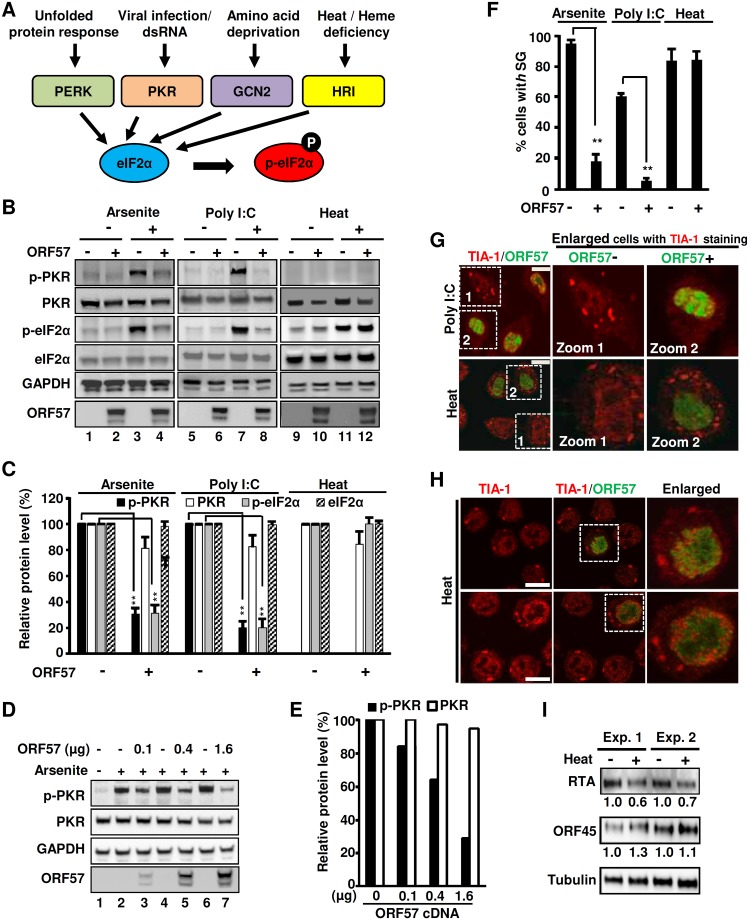
Viral ORF57 inhibits eIF2α phosphorylation through down-regulation of PKR pathway. (A) Diagrams of four major stress pathways leading to phosphorylation of eIF2α via different kinases. PERK, PKR-like endoplasmic reticulum kinase; PKR, protein kinase R; GCN2, general control nonderepressible 2; HRI, heme regulated inhibitory kinase. (B-C) ORF57 selectively inhibits the arsenite- or poly I:C-induced phosphorylation of both PKR and eIF2α, but not heat-induced phosphorylation of elF2α. HeLa cells transfected with an ORF57-Flag expressing vector or an empty Flag vector for 24 h were treated with arsenite, poly I:C or heat. The relative levels of p-eIF2α (Ser 51), phospho-PKR (Thr 451), total eIF2α, and total PKR in each sample were determined by Western blot with the corresponding antibodies and are plotted in bar graphs (C), with the expression level in the sample without ORF57 being set to 100% after normalized to GAPDH which served as a loading control. **P<0.01 in student *t*-test. (D-E) Dose-dependent decrease of PKR phosphorylation by ORF57. HeLa cells transfected with an ORF57-Flag expressing vector with an increasing dose (0.1, 0.4, 1.6 μg) or an empty Flag vector (1.6 μg) for 24 h were treated with arsenite. The relative levels of phospho-PKR (Thr 451), and total PKR in each sample were determined by Western blot with the corresponding antibodies and are plotted in bar graphs (E), with the expression level in the arsenite-treated sample without ORF57 being set to 100% after normalized to GAPDH which served as a loading control. (F-G) ORF57 inhibits SG formation induced by poly I:C, but not by heat. Bar graphs (F) show percentage of cells with SG counted from at least 100 cells in each group under different stress conditions in the absence or presence of viral ORF57. The error bar indicates mean ± SD from three independent experiments. **P<0.01 in Chi-squared test. A selective imaging field of HeLa cells from each induction condition is shown with TIA/ORF57 co-staining (G), along with two magnified images on individual cells. (H-I) Effect of heat-induced SG on viral gene expression. (H) Induction of SG during KSHV infection by heat. BCBL-1 cells at 8 h induction by VA were heat treated at 44°C for 40 min and then double stained with anti-TIA and anti-ORF57 antibodies. Bar = 10 μm. (I) Western blot analyses of viral protein expression during KSHV lytic infection in BCBL-1 cells with heat-induced SG formation. Total cell lysate of BCBL-1 cells induced by VA for 8 h and then heat treated at 44°C for 40 min were blotted by using anti-RTA or anti-ORF45 antibodies. The SG-altered change (%) in viral protein expression was calculated by protein band signal density of treated versus untreated samples after normalization to tubulin (loading control).

During cellular stress eIF2α can be phosphorylated by four different kinases, and which kinase is activated depends on the cause of stress (Fig 4A) [14]. Of particular interest, both viral infection [24,58] and arsenite [59] commonly activate PKR although GCN2 could be activated by Sindbis virus in a report [60]. PKR is well known for its antiviral activity by induction of interferon and is both a cytoplasmic and a nuclear dsRNA-binding protein [61,62]. We confirmed that arsenite did induce phosphorylation of PKR and SG formation in both HeLa and BCBL-1 cells, which could be specifically blocked by a PKR inhibitor (S4A–S4D Fig). These observations exclude the possibilities of other three pathways being involved in the studied SG formation in this report, although arsenite was also reported to mediate eIF2α phosphoryaltion through HRI in erythroid cells [63] and mouse fibroblast cells [64]. Virus infection activates PKR through the binding of viral dsRNA to the dsRNA-binding domain (RBD) of PKR, whereas arsenite activates PKR by inducing PACT to bind with and activate PKR [65,66]. PKR contains at least 15 autophosphorylation sites, but phoshorylation at both Thr 446 and Thr 451 is critical for its activation, and subsequent phosphorylation of eIF2α [67,68].

To elucidate the mechanism of ORF57-mediated inhibition of eIF2α phosphorylation, we investigated whether ORF57 could inhibit PKR activation and phosphorylation in HeLa cells under three different stress conditions (arsenite, double-stranded poly I:C, or heat stress) (Fig 4B). An optimized poly I:C dose was used to mimic dsRNA [69] to specifically activate PKR-mediated eIF2α phosphorylation (S5A Fig). Heat treatment at 44°C for 40 min served as an alternative route of cell stress resulting in eIF2α phosphorylation. As expected, both arsenite and poly I:C induced PKR phosphorylation along with eIF2α phosphorylation (Fig 4B, compare lanes 1 vs 3 and 5 vs 7). However, heat shock induced eIF2α phosphorylation without PKR phosphorylation. Thus, the heat shock-induced phosphorylation of eIF2α is not related to PKR activity (Fig 4B, compare lanes 9 vs 11) as has been reported in reticulocytes [63]. We did not see any induction in phosphorylation of PERK (PKR-like endoplasmic reticulum kinase) by arsenite (S5B Fig). Interestingly, we observed a dramatic reduction (~75%) of both phosphorylated PKR (p-PKR) and p-eIF2α when ORF57-expressing cells are exposed to either arsenite or poly I:C (Fig 4B, compare lanes 3 vs 4 and 7 vs 8, and Fig 4C), with only minimal or no change in overall PKR and elF2α protein levels. In contrast, ORF57-expressing cells exhibited no effect on the phosphorylation of eIF2α induced by the heat shock (Fig 4B, compare lanes 11 vs 12, and Fig 4C), but a dose-dependent, increased expression of ORF57 exhibited a steady decrease in PKR phosphorylation (Fig 4D and 4E). The ORF57 prevention of arsenite- and poly I:C-induced PKR phosphorylation with no change in total PKR levels was also observed in HEK293 cells (S5C Fig), indicating that the inhibitory effect of PKR phosphorylation by ORF57 is not cell-specific and could take place as early at 15 min of arsenite treatment, the earliest time point of the sample collection in this study (S5D Fig). Moreover, ORF57 was found to block the phosphorylation of TLR3 (toll-like receptor 3) induced by dsRNA (poly I:C) (S5E Fig) to activate Interferon regulatory factor 3 (IRF3) and production of type 1 interferon [70–73].

By IF microscopy, we found that both poly I:C and heat stress induce SG formation in HeLa cells (Fig 4F and S6 Fig), however, ORF57 was found to inhibit SG formation when induced either by arsenite or by poly I:C, but not when induced by the heat stress (Fig 4F and 4G and S6 Fig). The functional relevance of how ORF57 modulation of SG-formation impacts KSHV gene expression and replication was further explored in BCBL-1 cells. Because ORF57 does not affect heat shock-induced SG formation in BCBL-1 cells with lytic KSHV infection (Fig 4H), the effect of heat-induced SG formation on the expression of viral RTA, a viral replication and transcription activator and ORF45, a viral tegument protein, was investigated in the VA-induced cells. We found that 40-min heat shock-induced SG formation showed no effect on their RNA levels by RT-qPCR, but led to reduction of RTA protein expression by 30–40% in VA-induced BCBL-1 cells. The 40-min heat-shock had little effect on the expression of ORF45, a less-sensitive viral early gene to RTA transactivation [74] and a relative stable and abundant protein [75] (Fig 4I). Together with our previous findings [47], these results reveal that ORF57 inhibits PKR activation, disrupts the PKR-mediated phosphorylation of eIF2α and, therefore, blocks SG formation to promote viral gene expression.

### ORF57 interacts with PACT, PKR, PABPC1 and eIF4E, but not eIF2α, G3BP, TIA-1 and eIF4G1

**Fig 5:**
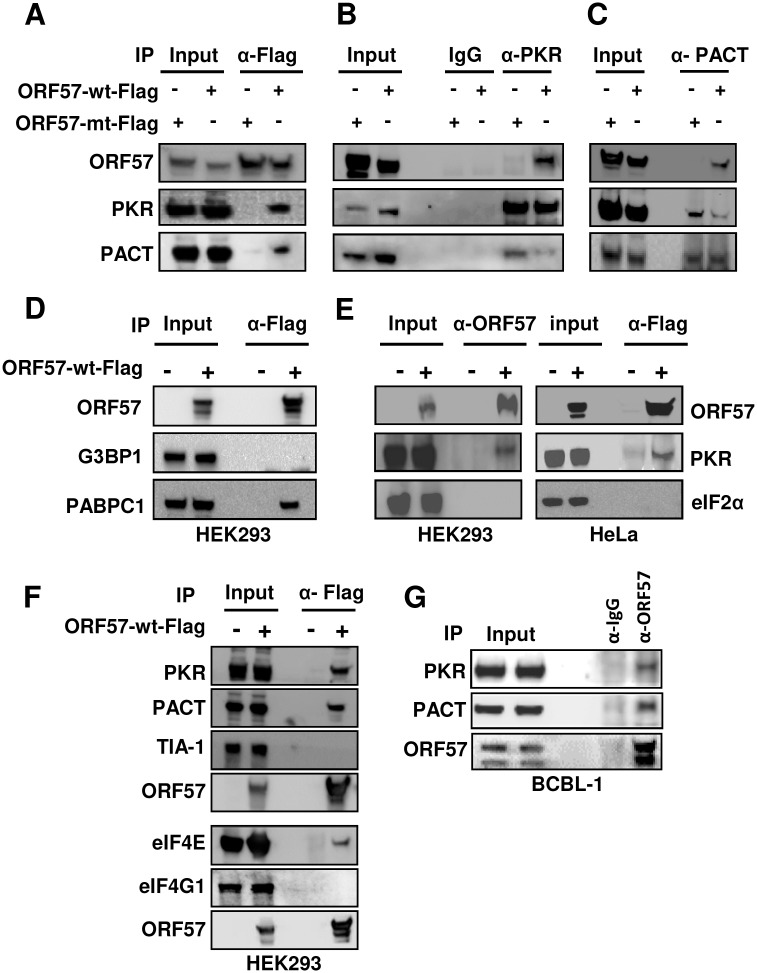
ORF57 interacts with PKR, PACT and PABPC1, but not G3BP1, eIF2α and TIA-1. (A-C) ORF57, but not ORF57 mt, interacts with components PACT and PKR of SG and inhibits their recruitment activities in arsenite-treated cells. HEK293 cells were transfected with an empty vector (-) or a vector expressing Flag-tagged ORF57-wt or ORF57-mt. Total cell lysates at 24 h after transfection were digested with RNase A/T1 to exclude any possibility of RNA-mediated protein-protein interactions in subsequent co-immunoprecipitation. (A) The ORF57-associated proteins were co-immunoprecipitated by a mouse anti-Flag antibody for ORF57 and the proteins in the pulldown were blotted with each corresponding antibody for endogenous PKR and PACT in addition to ORF57. (B-C) ORF57 interaction with PACT and PKR inhibits arsenite-induced PACT-PKR interactions. RNase-treated cell extracts in (A) were immunoprecipitated by an anti-PKR (B) or anti-PACT (C) antibody, and blotted for PKR-associated ORF57 and PACT (B) or PACT-associated ORF57 and PKR (C). Anti-Flag antibody was used to blot wt and mt ORF57. (D-F) Interaction of ORF57 with PABPC1, PKR, PACT and eIF4E, but not with G3BP1, eIF2α, TIA-1 and eIF4G1. The cell extract derived from HEK293 or HeLa cells with transfection of an empty vector or an ORF57-wt-Flag expression vector were treated with RNase A/T1 and then co-immunoprecipitated by a mouse anti-Flag for ORF57 (D-F) or a polyclonal rabbit anti-ORF57 (E, left panel) for ORF57-associated G3BP1 and PABPC1 (D), PKR and eIF2α (E), and PKR, PACT, TIA-1, eIF4E and eIF4G1 (F). Approximate 30% proteins pulled down from the co-IP were blotted with the corresponding antibody for G3BP1, PABPC1, PKR, eIF2α, PACT, TIA-1, eIF4E, eIF4G1 and ORF57, respectively. (G) Interaction of ORF57 with endogenous PACT and PKR in BCBL-1 cells with lytic KSHV infection. The cell extracts derived from VA-induced BCBL-1 cells for 24 h were treated with RNase A/T1 before being used for co-IP of endogenous PKR and PACT by a polyclonal rabbit anti-ORF57 antibody. A non-specific rabbit IgG was used in parallel as a negative control. Co-IPed PKR and PACT with ORF57 were detected by Western blot analysis using corresponding antibodies.

Given that both viral infection [24,58] and arsenite treatment [66] activate PKR and induce SG formation, and arsenite activates PKR by inducing PACT to bind PKR [65,66], we examined the mechanism by which ORF57 inhibits arsenite/poly I:C activation of PKR. By co-immunoprecipitation (co-IP) in combination with Western blot analysis we found that ORF57, but not the ORF57-mt, interacts with both PKR and PACT independent of RNA (Fig 5A–5C). Interestingly, the interaction of ORF57 individually with either PKR or PACT disrupts the interaction between PACT and PKR. As shown in Fig 5B and 5C, various co-IP experiments using an anti-PKR antibody (Fig 5B) revealed a remarkable reduction in the amount of PACT associated with PKR when ORF57 is present. Similarly, using an anti-PACT antibody (Fig 5C), we observed a significant reduction in the association of PKR with PACT in the presence of ORF57. This mechanistic function of KSHV ORF57 in blocking SG formation resembles that of the TRBP-PACT interaction and inhibition of PKR activation [76], but differs from other viruses that rely on cleavage or direct interaction with G3BP to block SG formation [28,33]. In this regard, ORF57 did not interact with G3BP (Fig 5D), nor alter G3BP expression (S2D Fig). In addition, ORF57 failed to interact with eIF2α (Fig 5E), TIA-1 or eIF4G1 (Fig 5F), although ORF57 did interact with PABPC1 [50] (Fig 5D) and eIF4E (Fig 5F), two common components of SG. Importantly, we further confirmed by co-IP and Western blotting that ORF57 interacts with both PACT and PKR in BCBL-1 cells during viral lytic infection (Fig 5G). Altogether, these studies indicate that PACT, PKR, PABPC1 and eIF4E are ORF57-interacting proteins and ORF57 binds to PACT and PKR and blocks PKR activation.

### ORF57 interacts with PACT via its two N-terminal RNA-binding motifs

**Fig 6:**
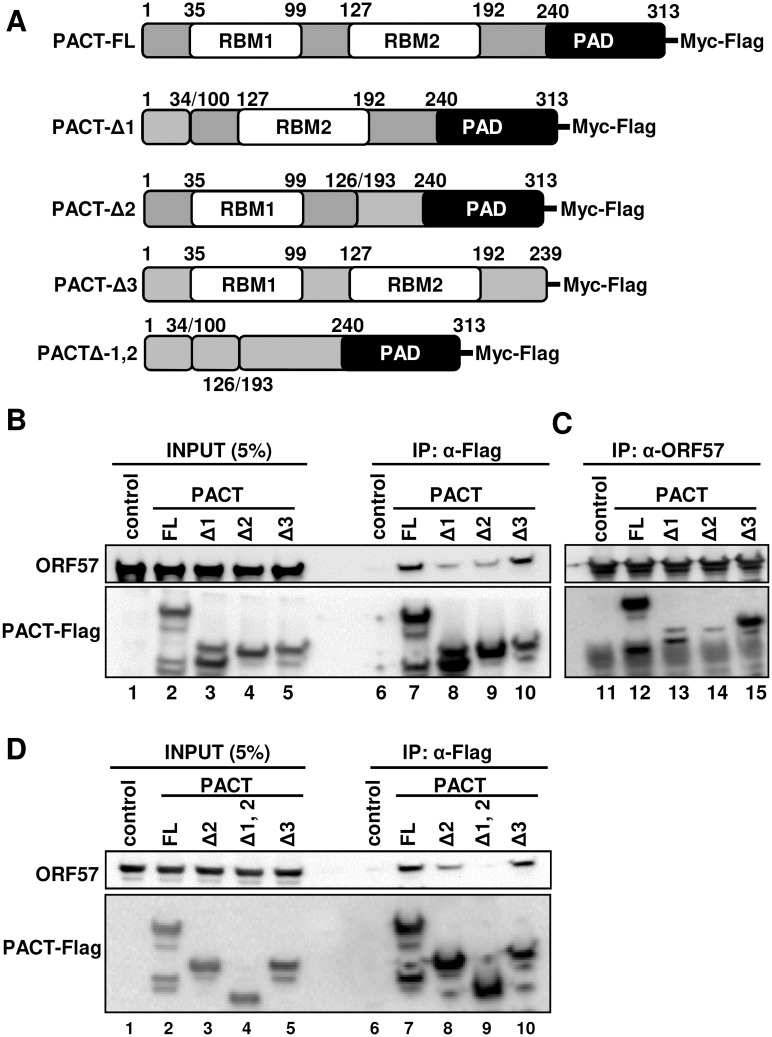
ORF57 interacts with PACT via its RBM1 and RBM2 motifs. (A) Schematic diagrams (not in scale) of full-length (FL) wt PACT and its deletion mutants. PACT contains two RNA-binding motifs (RBM1 and RBM2) and a PKR-activation domain (PAD). Numbers indicate the positions of amino acid (aa) residues for each domain in PACT. A series of PACT deletion mutants either with Δ1 missing RBM1 (aa 35–99), Δ2 missing RBM2 (aa 127–192), Δ3 missing PAD (aa 240–313) or double mutant Δ1,2 missing both RBM1 and RBM2) (aa 35–99 and aa 127–192) were generated by overlapping PCR. (B-D) Mapping of ORF57-PACT interacting domains. HEK293 cell extract containing ectopically-expressed Myc-Flag-PACT or its deletion mutant was mixed with the cell lysate containing untagged ORF57. The mixture was digested with RNase A/T1 and subjected to co-IP either with anti-Flag M2-coated beads (B, D) or with polyclonal rabbit anti-ORF57 antibody-coated beads (C). The proteins in the co-IP complex were detected by Western blot with the corresponding antibodies.

PACT contains two RNA-binding motifs (RBM) in its N-terminal half and a PKR-activation domain (PAD) in its C-terminal half. To determine the specific domain of PACT interacting with ORF57, the cell lysates containing individual Flag-PACT deletion mutants were mixed with the cell lysates containing ORF57 protein and followed by RNase A/T1 treatment to avoid any possible RNA-mediated protein-protein interaction in the subsequent anti-Flag antibody co-IP for PACT-associated ORF57 or anti-ORF57 antibody co-IP for ORF57-associated PACT. Western blot analysis of the proteins pulled down by the co-IP revealed that ORF57 interacts with PACT through its two RBM motifs and this interaction is independent of RNA. As shown in Fig 6, deletion of either RBM1 (PACT-Δ1) or RBM2 (PACT-Δ2) from PACT significantly reduced the binding of PACT to ORF57 (Fig 6B and 6C, compare lanes 7 and 12 to lanes 8–9 and 13–14), but deletion of the PAD (PACT-Δ3) did not (Fig 6B and 6C, compare lanes 7 and 12 to lanes 10 and 15). This study also demonstrated that further deletion of both RBM1 and RBM2 from PACT (PACT-Δ1,2) completely prevented the binding of PACT to ORF57 (Fig 6D, compare lane 9 to lanes 7–8 and 10). Based on these data, we concluded that ORF57 interacts with PACT via its two RBM motifs.

### ORF57 interacts with the N-terminal dsRNA-binding domain of PKR

**Fig 7:**
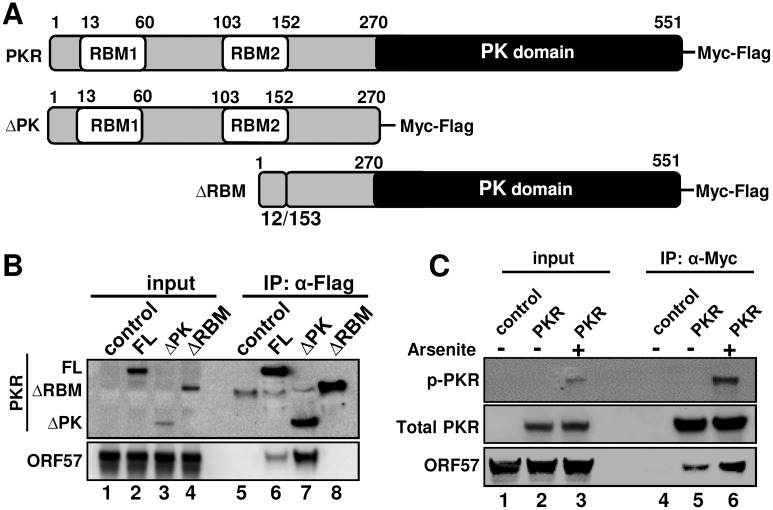
Viral ORF57 interacts with the RBM domain of PKR. (A) Schematic diagrams (not in scale) of PKR and its deletion mutants. RBM, dsRNA-binding motifs; PK domain, protein kinase domain. Numbers above each diagram represent amino acid positions in PKR protein (B) Mapping of ORF57-PKR interacting domains. HEK293 cell extract containing Myc-Flag-tagged full-length PKR or its deletion mutant ΔPK or ΔRBM was mixed with the cell extract containing untagged ORF57. The mixture was then digested with RNase A/T1 and immunoprecipitated with an anti-Flag antibody for PKR. The proteins in the pulldown were detected by Western blot using anti-ORF57 or anti-Flag for PKR. (C) ORF57 interacts more efficiently with phosphorylated PKR. HEK293 cells expressing full-length PKR-Myc-Flag were treated with arsenite for 30 min. Total cell extract was mixed with another HEK293 cell extract containing ORF57-Flag and digested with RNase A/T1. The PKR-ORF57 complex was pulled down with anti-Myc beads and blotted by anti-Flag antibody for total PKR, anti-phosphorylated PKR (Thr 451) for p-PKR and anti-ORF57 for ORF57.

PKR has a N-terminal regulatory domain containing two dsRNA-binding motifs (RBM1 & RBM2) and a C-terminal kinase domain (Fig 7A). To determine the specific domain of PKR interacting with ORF57, we generated two deletion mutants of PKR either by deletion of the kinase domain (ΔPK) or by deletion of the dsRNA-binding domain (ΔRBM). Both deletion mutants and the full-length (FL) PKR have a chimeric Myc-Flag tag and were individually expressed in HEK293 cells separately from untagged ORF57. By mixing the PKR cell extract with the ORF57 cell extract, followed by RNase A/T1 treatment to avoid any possible RNA-mediated protein-protein interaction in the subsequent anti-Flag or anti-Myc co-IP for PKR-associated ORF57, we found that the FL PKR and the N-terminal RBM domain (ΔPK), but not the kinase domain (ΔRBM) interact with ORF57 (Fig 7B, compare lanes 6–7 to 8 for ORF57). Interestingly, the ΔPK exhibited ~6.5-fold greater binding to ORF57 than did FL PKR (Fig 7B, compare lanes 7 to 6). Moreover, we found that the phosphorylated FL PKR increased its binding capacity toward ORF57 ~3 times more when activated in cells treated with arsenite (Fig 7C, compare lanes 6 to 5). Overall, we find that ORF57 interacts with the N-terminal RBM-containing domain of PKR, and the conformational change that occurs in PKR during activation allows ORF57 to bind to the N-terminus of PKR with greater affinity.

### ORF57 inhibits autophosphorylation of PKR by blocking dsRNA binding to PKR

**Fig 8:**
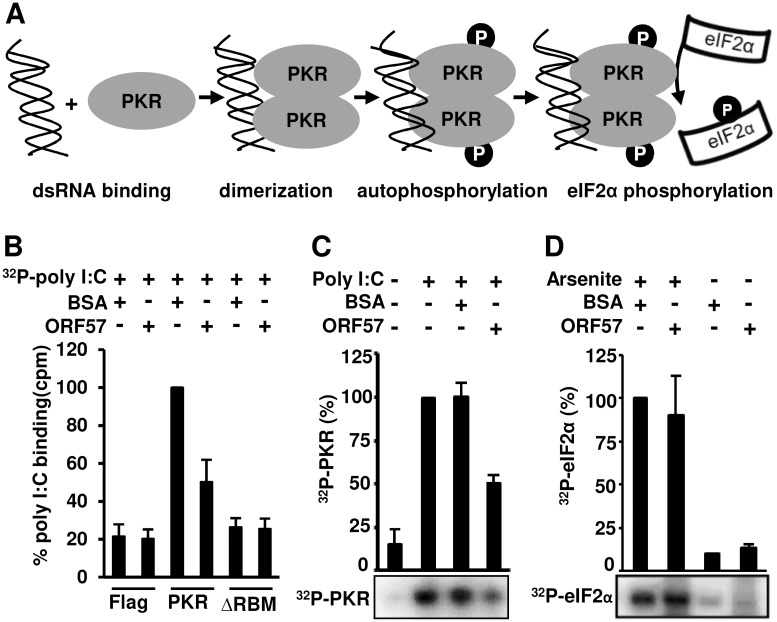
ORF57 inhibits autophosphorylation of PKR by blocking its interaction with Poly I:C, but does not directly affect elF2α phosphorylation. (A) Schematic diagrams of PKR activation. PKR binds to dsRNA poly I:C leading to its dimerization and autophosphorylation. The phosphorylated PKR at the kinase domain catalyzes eIF2α phosphorylation. (B) ORF57 interacts with PKR and prevents PKR from binding to poly I:C. Purified PKR-Myc-Flag or its mutant ΔRBM (Fig 7A) immobilized on Myc beads was incubated with ORF57 or BSA for 15 min at room temperature before addition of ^32^P-poly I:C. Bar graphs with means ± SD (n = 4, each in triplicate) show the binding of ^32^P-poly I:C to PKR or its ΔRBM mutant in the presence of ORF57 or BSA, quantified by scintillation count. Flag peptide addition to Myc beads served as a negative control for the Myc beads only (no immobilized PKR protein). (C) ORF57 inhibits PKR autophosphorylation. Purified PKR-Myc-Flag on Myc beads was used in the assay in the presence of [γ-^32^P-ATP], with or without poly I:C or with poly I:C plus ORF57 or BSA. The incorporated ^32^P into PKR due to autophosphorylation induced by poly I:C binding was measured by autoradiography of a SDS-PAGE gel after normalized to BSA control. The bar graph with means ± SD is derived from three independent experiments, with a representative SDS-PAGE gel image shown below the bar graph. (D) ORF57 doesn’t directly affect eIF2α phosphorylation. Inactive or arsenite-activated PKR-Myc-Flag immobilized on Myc beads was mixed with ORF57 or BSA protein and followed by addition of GST-eIF2α. An in vitro kinase reaction in the presence of [γ-^32^P-ATP] was carried out to phosphorylate GST-eIF2α. Incorporated ^32^P into GST-eIF2α was measured using SDS-PAGE and followed by autoradiography. The bar graph with means ± SD is derived from three independent experiments, with a representative SDS-PAGE gel image shown below the bar graph.

PKR activation depends on its binding to dsRNA via its two RBMs. This interaction induces PKR dimerization at the C-terminal kinase domain, which in turn leads to autophosphorylation of PKR (Fig 8A). Once phosphorylated, each subunit kinase domain in the dimerized PKR can independently phosphorylate the substrate eIF2α [14,77] (Fig 8A). To investigate the mechanism of how the ORF57-PKR interaction prevents phosphorylation of PKR and eIF2α, we first performed an in vitro competitive binding assay by which Myc-Flag-tagged PKR and its ΔRBM mutant immobilized separately on the anti-Myc beads were compared for competitive binding with ^32^P-poly I:C and recombinant ORF57. To do this, ^32^P-poly I:C was first mixed with recombinant ORF57 or BSA before allowing to interact with the immobilized PKR. As shown in Fig 8B, we observed that while BSA didn’t compete with ^32^P-poly I:C to interact with PKR, the recombinant ORF57 protein significantly reduced this interaction to ~50%. The PKR ΔRBM mutant and Flag control both served as negative controls, and ORF57 had no effect on their basal level of binding to ^32^P-poly I:C. These data suggest that ORF57 interacts with the N-terminal domain of PKR to prevent PKR binding to dsRNA.

We next examined that ORF57 prevention of PKR binding to dsRNA might affect PKR autophosphorylation. Subsequently, an in vitro autophosphorylation assay was conducted by incubation of recombinant ORF57 or BSA (a negative control) with immobilized PKR beads first before adding poly I:C and [γ-^32^P]-ATP. By examining the ^32^P-labelled PKR, we demonstrated that poly I:C did stimulate PKR autophosphorylation in the absence or presence of BSA, but this poly I:C induction of PKR autophosphorylation could be reduced by ~50% in the presence of ORF57 (Fig 8C).

In a separate experiment, we also tested whether ORF57 prevents the phosphoryated-PKR (p-PKR) from in turn phosphorylating its substrate eIF2α. To do so, we performed an in vitro kinase assay using a GST-eIF2α. The Myc-Flag-tagged PKR expressed in HeLa cells treated with or without arsenite was immobilized on anti-Myc beads and used to phosphorylate GST-eIF2α in the presence of recombinant ORF57 or BSA in a kinase reaction containing [γ-^32^P]-ATP]. By examining the amount of ^32^P-labelled GST-eIF2α, we found that while the inactive PKR from the cells without arsenite treatment did not exert much kinase activity on GST-elF2α, the arsenite-activated p-PKR did actively phosphorylate GST-eIF2α equally well both in the presence of BSA or ORF57 (Fig 8D). The inability of ORF57 to inhibit phosphorylation of GST-elF2α was not due to a lack of interaction between ORF57 and the active p-PKR. In fact, ORF57 was found to associate more efficiently with the activated p-PKR (Fig 7C). From these results presented above, we conclude that ORF57 interacts with the RBM motifs of PKR and prevents PKR binding to dsRNA or PACT and PKR activation by autophosphorylation, consequently preventing eIF2α phosphorylation and SG formation. However, once PKR is phosphorylated, ORF57 is unable to prevent p-PKR from phosphorylating eIF2α by its C-terminal kinase domain.

### KSHV vSOX, but not vIRF-2, inhibits arsenite-mediated SG formation

**Fig 9:**
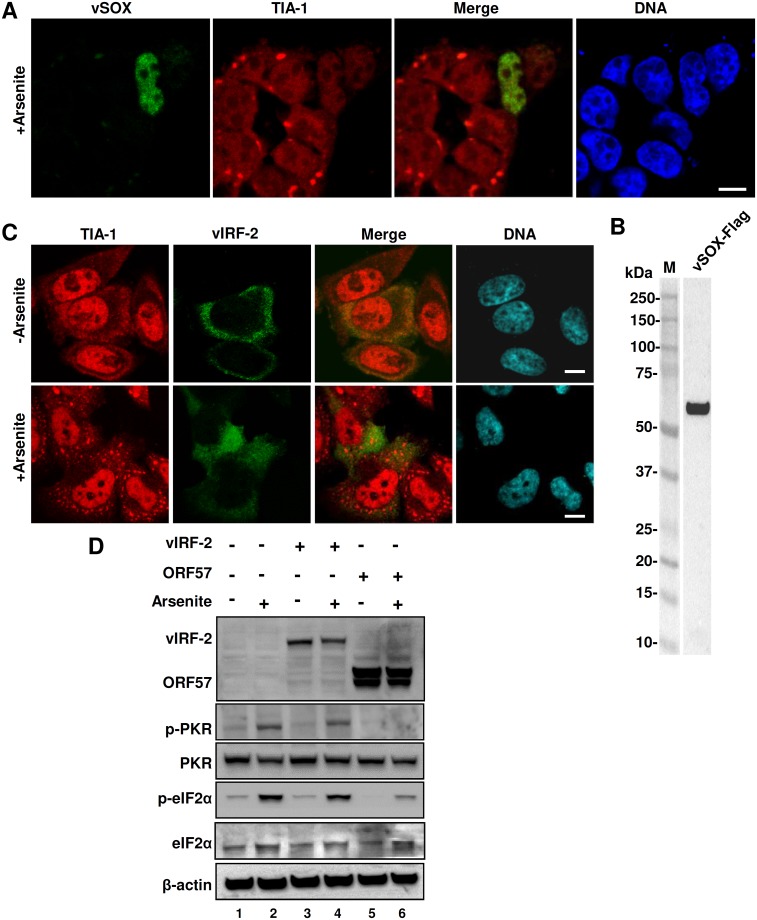
KSHV ORF37 (vSOX) inhibits arsenite-induced SG formation, but KSHV vIRF-2 does not. (A-B) KSHV ORF37 (vSOX) protein disrupts SG formation in HEK293 cells. HEK293 cells transfected with a vSOX-FLAG expression vector for 24 h were induced by arsenite for SG formation, fixed and stained with an anti-TIA-1 antibody (red) and an anti-FLAG antibody (green) for vSOX expression. The nuclei were counterstained with Hoechst dye (A). The cell lysates were blotted by an anti-FLAG antibody for vSOX protein (B). (C-D) KSHV vIRF2 does not inhibit SG formation and PKR phosphorylation. HeLa cells transfected for 24 h with an empty vector or a vector expressing KSHV vIRF2 or ORF57 were treated with arsenite to induce SG formation. Part of the transfected cells were used for IFA staining of SG-specific TIA-1 (red) in combination with anti-FLAG staining of vIRF2-Flag (green) (C) and other part of the cells were used for Western blotting (D). The nuclei were counterstained with Hoechst stain (C). Phosphorylated PKR (p-PKR) or eIF2α (p-eIF2α) in the cell lysate with or without arsenite treatment was blotted using a phosphor-specific PKR or eIF2α antibody and total level of PKR or eIF2α protein was blotted by an anti-PKR or anti-eIF2α antibody. The β-actin served as a loading control. Bar = 10 μm (A and C).

In the course of drafting our manuscript for publication, Finnen and colleagues reported that viral *vhs* protein encoded by UL41 in herpes simplex virus type 2 (HSV-2) suppresses SG formation [78]. Although α-herpesvirus vhs and γ-herpesvirus vSOX are not homologs, both are RNA endonucleases and exert their host shutoff function by digesting host mRNAs [79–82] which are fundamental for SG formation [8]. In assumption of KSHV vSOX encoded by viral ORF37 in blocking SG formation via a mechanism similar to HSV-2 vhs [78], we transfected both HEK293 and HeLa cells with a Flag-tagged KSHV vSOX expression vector for 24 h and stained the cells for TIA-1-specific SG formation in the presence or absence of vSOX after induction by arsenite for 30 min. We confirmed vSOX expression in HEK293 cells by anti-Flag antibody staining of HEK293 cells (Fig 9A) and by anti-Flag antibody Western blot (Fig 9B), but had difficulty to express vSOX in HeLa cells. As expected, HEK293 cells expressing no vSOX displayed SG formation induced by arsenite, but all cells expressing vSOX did not (Fig 9A). In contrast to ORF57, vSOX in transfected HEK293 cells had no effect on arsenite-induced phosphorylation of eIF2α. These results clearly indicate that KSHV vSOX inhibits SG formation in HEK293 cells, similar to HSV-2 vhs [78] by degradation of RNA [80–82].

Considering that KSHV interferon regulatory factor 2 (vIRF-2) might play an inhibitory role in PKR activation and PKR-mediated phosphorylation of eIF2α [83], we compared ORF57 with vIRF-2 in regulation of PKR activation and phosphorylation of eIF2α in arsenite-treated HeLa cells. Because the actual vIRF-2 ORF splits into two separate exons, with exon 1 in K11.1 and exon 2 in K11 [84] and encodes a full-length vIRF-2 having 680 aa residues, the annotated vIRF-2 ORF encoding 163 aa residues in an early report [83] based on initial ORF annotation in the KSHV genome [85,86] was not an authentic vIRF-2 ORF. Thus, we cloned a full-length vIRF-2 encoding 680 aa residues and expressed as a Flag-vIRF-2 in HeLa cells with or without arsenite treatment. As shown in Fig 9C, vIRF-2 was expressed predominantly as a cytoplasmic protein, but exhibited no effect on arsenite-induced SG formation. When compared with ORF57, we found that vIRF-2 in HeLa cells did not inhibit arsenite-induced PKR phosphorylation, nor eIF2α phosphorylation (Fig 9D, compare lane 4 to lane 3). In contrast, ORF57 at the same condition displayed the expected inhibition on phosphorylation of both PKR and eIF2α (Fig 9D, compare lane 6 to lane 2). According to these results, we conclude that the full-length vIRF-2 which modulates the host antiviral response [87–89] has no inhibitory function in activation of PKR pathway.

### PKR inhibits production of KSHV virions

**Fig 10:**
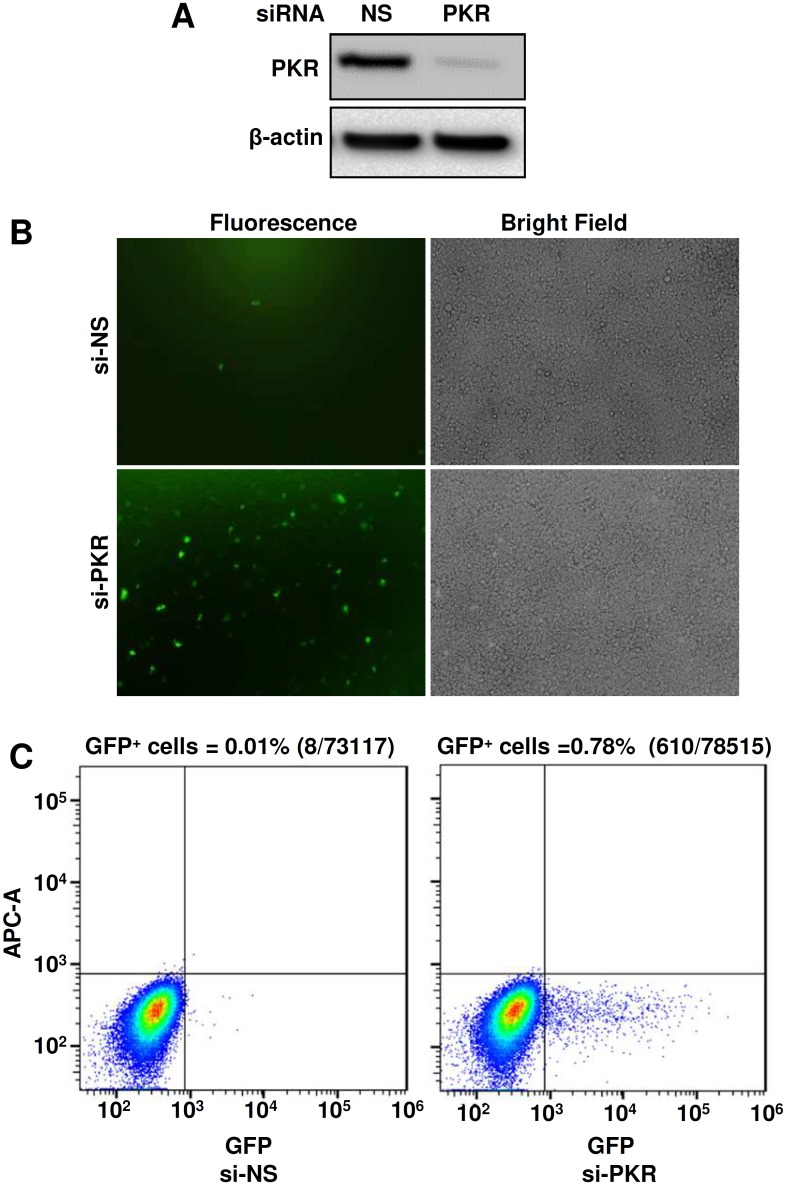
Knockdown of PKR expression increases production of KSHV virions. (A) Knockdown of PKR expression by a PKR-specific siRNA in iSLK-BAC16 cells by Western blot analysis. (B) Analysis of KSHV virus production after siRNA knockdown of PKR expression. KSHV replication in iSLK-BAC16 cells was induced by treatment with 1 mM sodium butyrate and 1 μg/ml doxycycline for 5 days. Supernatants obtained from the induced iSLK-BAC16 cells, which contain GFP-viruses, were used to infect HEK293 cells. The infected HEK293 cells were observed at 48 h infection for GFP expression as an indication of KSHV infection. (C) Virus-infected GFP-positive HEK293 cells were quantitated by FACS analysis. Each transfection/induction was performed in triplicate and three replicate infections were performed with each supernatant. Number of GFP-positive cells over the total number of cells in each infection was counted and expressed as a percentage (%). One representative of three infections is shown.

Previously, we and others demonstrated that KSHV ORF57 is essential for KSHV replication and virus production [45,90]. Our observations in this study showed that KSHV ORF57 inhibits PKR activation and disrupts the PKR-mediated phosphorylation of eIF2α to block SG formation (Fig 4B–4G). A well-recognized outcome of SG formation is to trigger the host cell antiviral response and inhibit virus production [91]. Therefore, we postulate that PKR might be a host inhibitory protein to block KSHV production and therefore, one of the ORF57 functions in blocking PKR activation and SG formation is to promote KSHV gene expression and virus production. To confirm this hypothesis, we examined KSHV virion production in a newly established iSLK-BAC16 cell line [92] with or without siRNA-mediated PKR knockdown. By using a PKR-specific siRNA, we found that efficient knockdown of PKR expression from iSLK-BAC16 cells (Fig 10A) resulted in significantly increased production of KSHV virions and led the iSLK-BAC16 culture supernatants being highly infectious for HEK293 cells (Fig 10B). Quantitative analyses by flow cytometry indicate that siRNA knockdown of PKR expression in iSLK-BAC16 cells led to ~78-fold increase of KSHV virion production over the cells with the normal level of PKR expression (Fig 10C).

### HSV ICP27, but not EBV EB2, exerts a similar function to ORF57 in inhibition of PKR/eIF2α/SG axis

**Fig 11:**
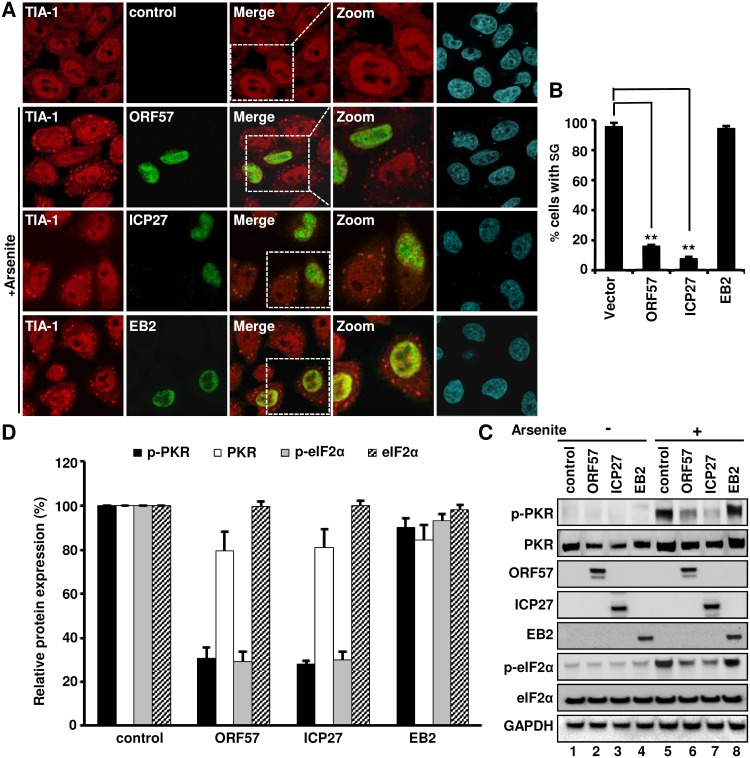
Differential function of KSHV ORF57 and its homologs in the herpesvirus family in inhibition of PKR-eIF2α pathway and SG formation. (A-B) HSV-1 ICP27, but not EBV EB2, inhibits SG formation in HeLa cells. HeLa cells expressing ORF57-Flag, ICP27-Flag, or EB2-Myc were treated with 0.5 mM arsenite for 30 min to induce SG. The cells were stained with anti-TIA-1 (Red) for SG, and anti-Flag (green) for ORF57 and ICP27 or anti-Myc (green) for EB2 (A). The nuclei were counterstained with Hoechst. Images were captured using confocal microscopy. Scale bar = 10 μm. Bar graph (B) shows at least 100 cells in each group with or without a viral protein being analyzed for SG-positive cells, with mean ± SD from three independent experiments. **P<0.01 in Chi-squared test. (C-D) HSV-1 ICP27, but not EB2, inhibits phosphorylation of endogenous PKR and eIF2α. HeLa cells at 24 h of transfection with each viral protein expressing vector or a Flag empty control vector were treated with 0.5 mM arsenite for 30 min and blotted with a corresponding antibody for each protein, anti-Flag for ORF57-Flag and ICP27-Flag and anti-Myc for EB2-Myc (C). GAPDH served as a loading control. The relative levels of p-PKR, PKR, p-eIF2α and eIF2α to GAPDH level were calculated over the amount of each protein from the vector control and shown in bar graphs (D), with mean ± SD from three independent experiments.

Given that HSV ICP27 and EBV EB2 (SM) proteins are homologues to ORF57, we reasoned that the ability to block the PKR/eIF2α/SG axis by KSHV ORF57 might be a conserved function in other herpesviruses. To investigate this possibility, we expressed ICP27 from HSV-1 and EB2 from EBV in HeLa cells and examined their influence on SG formation and phosphorylation of both PKR and eIF2α. The expression of ORF57, ICP27 or EB2 protein failed to induce SG in HeLa cells, but the three homologues exhibited a functional disparity in cells treated with arsenite. While both ORF57 and ICP27 abrogated the formation of SG in ~85% of arsenite-treated cells (Fig 11A and 11B), EB2 did not exert such an inhibitory function on SG formation (Fig 11A). By Western blotting of the lysates prepared from untreated or arsenite-treated cells transfected with an empty control vector or a vector expressing individual viral proteins, we found that both ICP27 and ORF57, but not EB2, inhibited the phosphorylation of ~70% PKR and ~75% eIF2α (Fig 11C, lanes 6–7 vs lanes 5–8 and Fig 11D), along with only a minimal effect on total PKR or eIF2α protein levels (Fig 11C and 11D). Altogether, these data indicate that the ability to inhibit SG formation in herpesviruses is conserved in HSV-1 through the ORF57 homologue ICP27.

## Discussion

Virus infection inevitably induces host cell stress. Thus, SG formation in the infected host cells has been widely appreciated as an antiviral defense mechanism [91,93]. SG formation is a downstream event of eIF2α phosphorylation which stalls translation initiation [10,94]. Of the four cellular kinases which can phosphorylate eIF2α and induce SG formation [77], PKR is a major player during viral infection. Viral dsRNA activates PKR and, therefore, induces SG formation. Although counteracted by many RNA viruses, regulation of the PKR/eIF2α/SG pathway by DNA viruses is poorly understood. Besides its activation by viral dsRNA, PKR is activated by PACT during arsenite stress [65,66]. Here, we utilized arsenite stress to explore the differential ability of KSHV infection to block SG in B cells and HEK293-derived Bac36 cells with a wt or ORF57-null (Δ57) KSHV genome. We found that KSHV, a DNA virus expressing the viral early protein ORF57, confers the infected B cells and Bac36-wt cells refractory to SG induction during lytic infection. Viral ORF57 alone blocks activation and phosphorylation of PKR and thereby SG formation. KSHV vSOX bearing intrinsic endoribonuclease activity also affects SG formation by degrading RNA. Other KSHV-encoded proteins examined in this study, such as RTA, ORF45, ORF49, LANA and vIRF-2, have no such function.

TIA-1 protein is a robust marker of SG and the N-terminal RRM domain TIA-1 binds to targeted RNA transcripts that have an AU-rich or C-rich element in the 5’ or 3’ UTRs [95–97]. TIA-1 nucleates SG formation via its C-terminal glutamine-rich prion-related domain (PRD) responsible for self-association [18]. Consistently, we found TIA-1 enrichment in the insoluble cell pellets during arsenite treatment, but this enrichment is prevented when ORF57 is present. The ORF57-mediated reduction of TIA-1 in the pellet is most likely a consequence of ORF57 inhibiting SG formation, but why a proportion of ORF57 resides in the insoluble pellets remains unknown, presumably being associated with ribosomes, microtubes, or other cellular debris. ORF57 does not interact directly with TIA-1 or G3BP1, another SG nucleator in addition to TIA-1 [98] and therefore, must indirectly influence the biochemistry of TIA-1 and G3BP1. Unlike TIA-1 and G3BP1, ORF57 does bind to PABPC1 and eIF4E, two important components of SG. ORF57 interaction with PABPC1 reduces the cytoplasmic pool of PABPC1 by promoting the redistribution of PABPC1 to the nucleus [50]. Although an important component of SG, PABPC1 is viewed as a passenger and does not function directly in SG formation [10,99], but may influence indirectly the ability of TIA-1 and G3BP1 to form SG. We also find that ORF57-mediated inhibition of SG formation is accompanied by a significant reduction in the amount of cytoplasmic PABPC1. PABPC1 binds to the 3’ poly(A) tail of eukaryotic mRNAs, and its interaction with the N-terminus of eIF4G stabilizes RNA and promotes both ribosome recruitment and translation initiation [100–102]. Whether the reduction in cytoplasmic PABPC1 mediated by ORF57 affects recruitment of the polyadenylated RNA transcripts by TIA-1 into SG needs to be investigated. Cap-binding protein eIF4E is a translation initiation factor which binds mRNA’s 5’ cap and mediates the cap structure of mRNA directly binding to the 40S ribosomes. Similar to PABPC1, eIF4E could be a passenger protein and does not function directly in SG formation, although ORF57 interaction with eIF4E, but not with eIF4G1, might be involved in regulation of protein translation initiation. The inability of ORF57 to block heat stress-induced SG formation indicates that the ORF57 interaction with PABPC1 and eIF4E is insignificant with regards to SG formation, at least under conditions of heat shock-induced cell stress.

**Fig 12:**
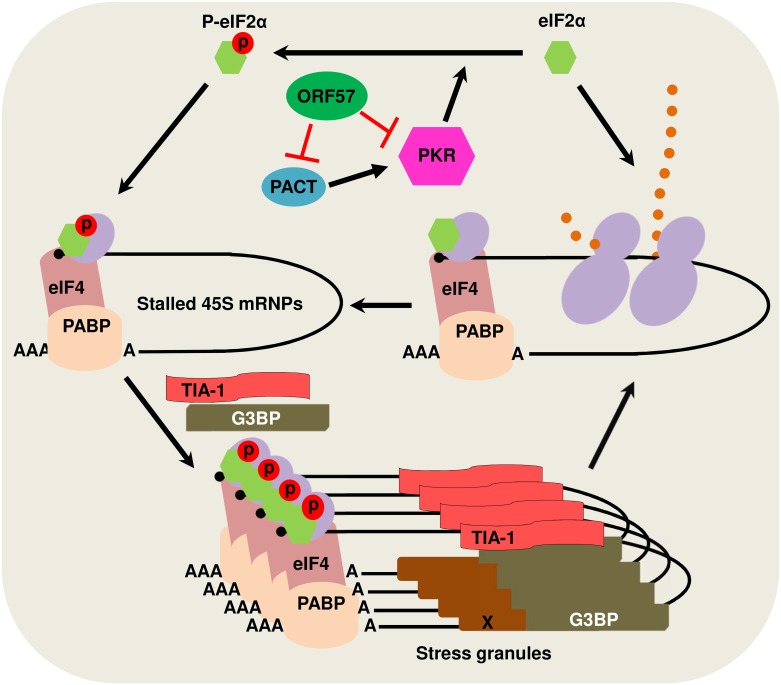
A schematic model showing KSHV ORF57 interactions with PACT and PKR in blocking phosphorylation of PKR and elF2α and inhibition of SG formation. In this model, ORF57 exerts no direct effect on eIF2α, nor its phosphorylation once PKR becomes activated and phosphorylated. Orange dots, elongating polypeptide chain from translating polysomes; X, other protein factors in stress granules.

KSHV ORF57 is an RNA binding protein and a posttranscriptional regulator of viral RNA transcripts [47]. Our finding that ORF57 functions as an inhibitor of PKR/eIF2α phosphorylation is intriguing and surprising. Further characterization of this inhibitory function of ORF57 led us to discover that KSHV ORF57 interacts with both PACT and PKR via their RBM motifs to prevent activation and phosphorylation of PKR and thereby, to inhibit eIF2α phosphorylation and SG formation (Fig 12). PKR is a dsRNA-binding protein important for the antiviral action of IFN and is a major cellular kinase that controls translation by phosphorylation of eIF2α [14]. We demonstrated that knocking down PKR expression in iSLK-BAC16 cells significantly promoted KSHV lytic infection and virion production. PKR is activated during viral infections [24,58] and experimentally by arsenite treatment [59]. The activation mechanisms are slightly different in that, viral infections activate PKR through the binding of viral dsRNA to the RNA-binding motif of PKR [103–105], whereas arsenite activates PKR via PACT, a PKR activating protein which heterodimerizes with PKR and activates PKR in the absence of dsRNA [65,66]. When we experimentally induce PKR phosphorylation by either arsenite treatment or incubation with poly I:C, the presence of ORF57 blocks PKR phosphorylation by binding directly to both PACT and PKR leading to a reduction in PACT-PKR and dsRNA (poly I:C)-PKR interactions. Other viral proteins, including TRS1 of cytomegalovirus [106] and UL41 of HSV-2 [36,37], exhibit a similar function in blocking PKR phosphorylation and activation to block SG formation. Even though ORF57 interacts with the N-terminal RBM domain of PKR and blocks it’s binding to dsRNA and its autophosphorylation, it has no direct effect on phosphorylation of eIF2α once PKR is activated and autophosphorylated and does not interact with eIF2α. The enhanced interaction of ORF57 with the p-PKR might be a result of conformational changes in p-PKR. This mechanistic function of ORF57 resembles that of TRBP which inhibits PKR activity through the interaction with PKR RBMs [76,107], but differs from poliovirus 3C protease which cleaves G3BP1 [28], SFV nsP3 and HSV-2 ICP8 which suppress SG formation by their FGDF motifs interacting with G3BP1 [33], and HSV-2 vhs which requires its endoribonuclease activity in disruption of SG formation [78]. KSHV ORF57 does not have a FGDF motif or interact with G3BP1 and bears no endoribonuclease activity. Although α-herpesvirus vhs and γ-herpesvirus SOX are not homologs, both are RNA endonucleases that digest host mRNAs [79,81]. Thus, it is not to our surprise that KSHV vSOX is capable of blocking SG formation by a mechanism similar to HSV-2 vhs [78]. Moreover, poxviruses induce a SG-like antiviral granule formation which does not entirely depend on PKR or eIF2α [108,109] and can be induced in the cells lacking eIF2α [109] or TIA-1 [110], although vaccinia virus E3L protein antagonizes PKR function and blocks the antiviral granule formation [110].

The discrepancy between EBV EB2 (SM) and both KSHV ORF57 and HSV-1 ICP27 in regulating the phosphorylation of PKR and eIF2α is another interesting result described in this report. KSHV ORF57, HSV-1 ICP27 and EBV EB2 are three well-known homologous proteins in regulation of viral RNA biogenesis at various lytic stages of viral infections. Although ORF57 deviates from ICP27 in protein structure and the function of RNA splicing, EBV EB2 is a more closely matched homologue to ORF57, with many functional similarities [47]. Here we show that both ICP27 and ORF57, but not EBV EB2, inhibits arsenite-induced SG formation by blocking phosphorylation of PKR and eIF2α (Fig 11). EBV EB2 was described as a dsRNA-binding protein and inhibits PKR activation via its RXP triplet repeats [111], but this domain with the RXP triplet repeats doesn’t exist in EB2 homologs and the function of EB2 in promoting protein translation appears independent of the PKR pathway [112]. ICP27 has not been characterized as a protein being able to block SG formation, but does appear in spontaneous SG during virus infection [36,37] and inhibits IFN signaling [113]. Our data show an extreme similarity of HSV-1 ICP27 with KSHV ORF57 in preventing SG formation by blocking phosphorylation of PKR and eIF2α, providing evidence for ectopic ICP27 being directly involved in regulating the PKR pathway in the absence of HSV infection.

KSHV encodes many proteins that function to evade the host immune system in multiple ways. Most of these viral proteins are homologous to cellular proteins and interfere with both innate and adaptive immune responses. In particular, immune evasion of the interferon pathway and the TLR pathway by KSHV vIFNs and other host mimics is one of the important strategies for KSHV to escape from the host innate immune response [114]. In this regard, KSHV vIRF-1, -2, or -3 blocks TLR3-mediated activation of IFN-responsive promoter activity [73] and vIRF1 decreases phosphorylation and nuclear translocation of IRF-3 in response to TLR3 activation [73]. Although a non-existing short-form vIRF-2 of KSHV was found to interact directly with PKR and inhibit PKR autophosphorylation and eIF2α phosphorylation [83], the full-length vIRF-2 in our study does not exhibit such a function or inhibition of SG formation. In addition, we found ORF57 also blocks poly I:C-induced phosphorylation of TLR3. Together with the finding that KSHV ORF57 regulates PKR pathway to suppress SG formation and PKR inhibits KSHV production, our data provide the first evidence that KSHV ORF57 plays a critical role in modulation of PKR/eIF2α/SG axis to enhance KSHV lytic infection.

## Material and methods

### Cell cultures and virus reactivation

Human HEK293 and HeLa cells (ATCC, Manassas, VA) were cultivated in DMEM (Thermo Fisher Scientific, Waltham, MA) supplemented with 10% fetal bovine serum (FBS, GE Healthcare, Logan, UT). Primary effusion lymphoma BCBL-1 cells (KSHV^+^) [55] obtained from the AIDS Research and Reference Reagent Program, Division of AIDS, NIAID, NIH, were grown in RPMI 1640 (Thermo Fisher Scientific) containing 10% FBS. KSHV lytic infection in BCBL-1 cells was reactivated by 1 mM sodium valproate (cat. no. P4543, Sigma-Aldrich, St. Louis, MO) for 24 h. HEK293-derived Bac36 cell lines stably harboring a wt KSHV genome (Bac36-wt) or an ORF57-null KSHV genome (Bac36-Δ57) were established in our lab as described [45]. KSHV lytic infection in Bac36 cells was reactivated with 3 mM sodium butyrate (cat. no. B5887, Sigma-Aldrich) for 24 h. All plasmid transfections were performed using LipoD293 transfection reagent (SignaGen Laboratories, Gaithersburg, MD) according to the manufacturer’s instruction. Unless indicated, for IFA and Western analysis, HeLa (2.5 ×10^5^) and HEK293 (5 × 10^5^) cells were plated a day prior to 1 μg (2 μg for vSOX) of plasmid DNA transfection in a 6-well plate. For IP, HeLa (2 × 10^6^) and HEK293 (5 × 10^6^) cells were plated a day prior to 5 μg plasmid DNA transfection in a 10-cm Petri dish.

### Antibodies and chemicals

The custom made rabbit polyclonal and mouse monoclonal anti-ORF57 antibodies were described earlier [45]. Mouse monoclonal and rabbit anti-RTA and mouse monoclonal anti-ORF45 antibodies were kindly provided by Drs. Yoshi Izumya and Fanxiu Zhu, respectively. Mouse monoclonal anti-β-tubulin (cat. no. T5201), anti-Flag M2 (cat. no. F1804), anti-c-Myc (9E10, cat. no. M4439) and rabbit polyclonal anti-Flag antibody (cat. no. F7425) were obtained from Sigma-Aldrich. Rabbit polyclonal antibodies anti-eIF2α (cat. no. 9722S) and anti-phospho-eIF2α (ser51) (cat. no. 9721S) and rabbit monoclonal anti-GAPDH (cat. no. 2118), anti-phospho-PERK (Thr 980) (16F8, cat. no. 3179) and anti-PERK (C33E10, cat. no. 3192) were obtained from Cell Signaling Technology. Other antibodies used were: rabbit polyclonal anti-TLR3 antibody (phospho-Tyr759) (cat. no. LS-C19344, LifeSpan BioScience Inc. Seattle, WA), anti-PABPC1 (cat. no ab21060, Abcam, Cambridge, MA), anti-PACT/PRKRA (cat. no 10771-AP, ProteinTech group, Rosemont, IL), anti-phospho (Ser 209)-eIF4E (cat. no. ab47605, Abcam), and anti-phospho PKR (pThr451) (cat. no. 527460, EMD Millipore, Billerica, MA); mouse monoclonal anti-eIF2AK2 (PKR) (cat. no. H000005610-M01, Abnova), anti-eIF4E (cat. no. 610269, BD Biosciences, San Jose, CA), anti-PACT/PRKRA (cat no. H00008575-M01, Abnova, Taipei, Taiwan), and anti-eIF4G1 (cat. no. ab54970, Abcam); goat polyclonal anti-TIA-1 (cat. no. sc-1751, Santa Cruz Biotechnology, Dallas, Texas), and rat monoclonal anti-HHV-8 LANA (cat. no. MABE1109, EMD Millipore). The peroxidase-conjugated secondary antibodies used in Western blotting were obtained from Sigma-Aldrich and all Alexafluor-conjugated secondary antibodies used in IFA were purchased from Thermo Fisher Scientific (Waltham, MA). Sodium arsenite (cat. No 38150), valproic acid sodium salt (cat. no. P4543), sodium butyrate (cat. no. B5887) and cycloheximide (cat. No. C-7698) were obtained from Sigma-Aldrich. PKR inhibitor (PKR_i_, cat. no. 527451) and its negative control (PKR_c_, cat. no. 527455) were obtained from EMD Millipore.

### Expression vectors and construction of plasmids

The expression vectors were used to express recombinant proteins: KSHV ORF57 (pcDNA-ORF57), ORF57-FLAG (wt, pVM7; mt NLS 2+3, pVM89), ORF57-GFP (wt, pVM8; mt NLS 2+3, pVM36), KSHV ORF59-FLAG (pVM18), pKY15 (HSV1-ICP27-FLAG) and pGS113 (myc-EBV-EB2) [48,52,57]. Empty pcDNA 3.0 and pCMV-FLAG 5.1 (Sigma-Aldrich) were used as a negative control. KSHV vSOX (ORF37) ORF was amplified by PCR using a primer pair of oVM400 (5’-CCGGAATTCACC/ATGGAGGCCACCCCCACAC-3’, nt 57273–57291) and oVM401 (5’-ACTGTCTAGA/CGGGCTGTGAGGGACGTTTG-3’, nt 57291–57273) on total DNA from BCBL-1 cells. The resulting PCR product was cloned into pFLAG-CMV-5.1 vector (Sigma-Aldrich) via *EcoRI* and *XbaI* sites to create plasmid pVM116. The identity of inserted ORF was verified by Sanger sequencing. The expression of vSOX-Flag fusion protein (486 aa + Flag tag, ~52 kDa) was confirmed by Western blot using anti-Flag antibody upon transfection in HEK293.

Full-length Protein Kinase R encoding plasmids (p-CMV-Entry, PKR-Myc-Flag or pPKR-FL) (cat. no., RC210792) was obtained from Origene (Rockville, MD). PKR deletion mutants (ΔRBM, aa 1-12/153-551) was generated by overlapping PCR using following primers: oVM78 5’-CCGTTGACGCAAATGGGC-3’ and oNS1 (5’-TGCTTCCTGTTT/CTCCATGAAGAAACCTGC-3’; PKR nt 1487-1476/1064-1047) for first PCR and oNS2 (5’-TTCTTCATGGAG/AAACAGGAAGCAAAACAATT-3’; PKR nt 1053-1064/1476-1495) and oNS3 (5’-CATCACTGGTCTCAGGATC-3’; PKR nt 2043–2025) for second PCR. Obtained two PCR products were combined and re-amplified with oNS3 and oVM78. The final PCR product was used to replace full length PKR using *Asp*718 and *Bcl*I sites in pPKR-FL plasmid. The PKR ΔPK mutant (aa 1–270) was generated by PCR using oVM78 and oNS4 (5’-AGTATTACGCGTATCCATGCCAAACCTCTTG-3’, PKR nt 1826–1808) as forward and reverse primers, respectively. The resulting PCR fragment was cloned to replace FL-PKR at *Bam*HI and *Mlu*I sites in PKR-FL plasmid.

Full-length PACT-Myc-Flag encoding plasmid (pPACT-FL) was obtained from Origene. Deletion of PACT-RBM1 (Δ1, aa 1-34/100-313), RBM2 (Δ2, aa 1-126/193-313) or both RBM1 and RBM2 (Δ1,2) was generated by overlapping PCR on pPACT-FL using following primers: oVM78 5’-CCGTTGACGCAAATGGGC-3’ in the CMV IE promoter region and oNS16 (5’- TGCATTGGCTTT/TGTTTTCCCTGGCTTAGCT-3’; PACT nt 1337-1326/1130-1112) for first PCR and oNS17 (5’-AGCCAGGGAAAACA/AAAGCCAATGCAAGTATTT-3’, PACT nt 1117-1130/1326-1344) and oNS18 (5’-CACTGGAGTGGCAACTTC3-3’; PACT nt 2166–2149) for second PCR were used for deletion of RBM1; oVM78 and oNS19 (5’-AGAAATATTACT/ATTAAGCTGGTTCTTTGGT3’-3’; PACT nt 1616-1605/1406-1388) for the first PCR and oNS20 (5’-AGAACCAGCTTAAT/AGTAATATTTCTCCAGAGA-3’; PACT nt 1393-1406/1605-1623) and oNS18 for the second PCR were used for deletion of RBM2. Obtained two PCR products in oligo-mediated deletion of RBM1 or RBM2 were combined and re-amplified with oVM78 and oNS18. The final PCR product (PACT-Δ1 or– Δ2) was used to replace full length PACT using *Bam*HI and *Mlu*I sites in the plasmid pPACT-FL. To make the PACT-Δ1,2 (aa 1-34/100-126/193-313), oVM78 and oNS16 were used for the first PCR and oNS17 and oNS18 were used for the second PCR on plasmid PACT-Δ2. The overlapped PCR products from oVM78 and oNS18 were cloned as above. The PACT- ΔPAD mutant (Δ3, aa 1–239) was generated from the parent pPACT-FL plasmid by PCR using oVM78 and oNS21 (5’-AGTATT/ACGCGT/TGTATTTGGAATACTAAGGA-3’, PACT nt 1745–1726) as forward and reverse primers, respectively. The resulting PCR fragment was cloned to replace the PACT-FL into the parent plasmid pPACT-FL using *Bam*HI and *Mlu*I sites.

Because vIRF2 is a split gene in the KSHV genome [84], the overlapping PCR was also performed by using oVM386 (5’-TACTCAGAATTCACC/ATGCCTCGCTACACGGAGT-3’, vIRF2 nt, 94127–94109) and oVM389 (5’- TCGCTCTGTGACCGTGATGAA-3’, vIRF2 nt 93435–93453) for the first PCR and oVM388 (5’-AGTCGCCACGCCCACAACAT-3’, vIRF2 nt 93496–93472) and oVM387 (5’-ATCGTGGATCC/GTCTCTGTGGTAAAATGGG-3’, vIRF2 nt 93435–93453) for the second PCR on cDNA derived from VA-activated BCBL-1 cell total RNA. Following by annealling the two PCR products and re-amplification using oVM386 and oVM387 to generate a spliced isoform of full-length vIRF2 in size of 2161 bps, the final PCR product was digested by *Eco*RI and *Bam*HI and cloned into p-Flag-CMV-5.1. The resulting plasmid was subsequently named as pVM105. All plasmids were verified by restriction digestion and sequencing.

### Western blot

Unless indicated otherwise, protein samples for Western blot were prepared by direct lysis of the cells in 2 × SDS sample buffer (Quality Biological) containing 5% 2-mercaptoethanol (Sigma-Aldrich). Samples were resolved on a 4%-12% SDS-PAGE gel in 1 × MOPS buffer (Thermo Fisher Scientific). The signal was detected with SuperSignal West Pico or Femto Chemiluminescent Substrate (Thermo Fisher Scientific).

### Induction of cellular stress

Sodium arsenite (cat. No. 38150, Sigma-Aldrich) solution (0.83 M) was prepared in water to serve as a 1660× stock solution. To induce oxidative stress the cells were cultivated in fresh culture medium containing 0.5 mM of sodium arsenite for 30 min. To mimic the cellular stress induced by dsRNA, double-stranded poly I:C (lyophilized polyinosinic–polycytidylic acid sodium salt) obtained from Sigma-Aldrich (cat. no. P0913) was dissolved in sterile DEPC-treated H_2_O containing 0.98% NaCl to make 5 mg/ml stock solution. Before use, poly I:C was denatured by incubation at 50°C water bath for 20 min followed by slowly cooling to room temperature for at least 45 minutes for proper annealing. The size of poly I:C was determined on a 1% agarose gel and was in size of ~250–300 bp. The amount of poly I:C required for induction of eIF2α phosphorylation was experimentally determined by transfection of HeLa cells with 0.1–5 μg of annealed poly I:C using Lipojet transfection reagent (SignaGen). To study inhibitory effect of ORF57 on PKR activation by poly I:C, HeLa or HEK293 cells with or without ORF57 expression were transfected with 1 μg poly I:C and incubated additional 8 h before harvesting. The heat stress was induced by incubation of cells at 44°C for 40 min. Before harvesting, the cells were washed with 1 × phosphate-buffered saline (PBS, Thermo Fisher Scientific).

### Chemical inhibition of PKR phosphorylation

The activation of PKR was inhibited by pre-incubation of cells for 1 h with increasing dose (1, 10 and 100 μM) of PKR inhibitor (PKR_I_) or PKR inhibitor control (PKR_C_) dissolved in DMSO. DMSO alone was used as a negative control. Cells were subsequently washed and incubated with fresh medium containing 0.5 mM of sodium arsenite for 30 min before harvesting.

### Inhibition of viral gene expression by heat stress

BCBL-1 cells (2 × 10^6^ cells) were induced with 1mM VA for 8 h followed by 40 min incubation at 44°C. The cells cultivated at 37°C were used as negative controls. Subsequently, the cells were harvested for preparation of total proteins in SDS sample buffer and total RNA extracted by TRIzol. Quantitative real-time RT-PCR (RT-qPCR) was performed using customized TaqMan probes and TaqMan Gene Expression Master Mix (Thermo Fisher Scientific) with standard 2-Δ(*ΔCT*) protocol.

### Stress-induced soluble and insoluble fractionation of TIA-1

HeLa cells with or without ORF57 expression were treated with 0.5 mM arsenite for 30 min to induce SG. After washing with ice-cold 1 × PBS, cells were lifted from plates using a cell scrapper and in RSB-200 buffer (10 mM Tris-HCl [pH 7.5], 200 mM NaCl, 2.5 mM MgCl_2_, 0.1% NP-40) supplemented with 1 × protease and phosphatase inhibitor cocktails (Roche) followed by sonication using 10 strokes at level 4 on a sonic dismembrator (Model 100, Fisher Scientific). The cell lysates were incubated on ice for 15 min and centrifuged at 15871 × g for 15 min at 4°C to separate soluble (supernatants) and insoluble (pellets) fractions. The cells without arsenite treatment were used as a negative control.

### Immunoprecipitation (IP)

IP was performed as described earlier [50]. Briefly, ectopic expression of proteins was obtained by individual plasmid transfection in HeLa or HEK293 cells grown in a 10-cm plate. Cells with or without plasmid transfection were washed with 1× PBS, lysed in 500 μl of 1× RSB-200 lysis buffer (10 mM Tris-HCL [pH 7.5], 200 mM NaCl, 2.5 mM MgCl_2,_ 0.1% NP-40 and protease inhibitor cocktail [Roche]), sonicated (10 strokes at level 4) and cleared by centrifugation at 11500 × g for 10 min at 4°C. In the assays designed to see the interaction of multiple overexpressed proteins, 100 μl total cell extract containing one overexpressed protein was mixed with 100 μl total cell extract with another overexpressed protein. The mixed cell lysates (200 μl) were then incubated with 5 μl of RNase A/T1 mixture containing 1.25 U RNase A and 50 U RNase T1 (Thermo Fisher Scientific) for 10 min at room temperature followed by pre-cleaning with pre-washed sepharose CL-4B beads (Sigma-Aldrich). The pre-cleaned cell lysates were mixed with 80 μl of antibody-coated protein A/G beads (50% slurry, Sigma-Aldrich) in 1 ml of IP buffer (50 mM HEPES [pH 7.5], 200 mM NaCl, 1 mM EDTA, 2.5 mM EGTA, 10% glycerol, and 0.1% NP-40, 1 × Roche’s protease inhibitor cocktail) and incubated overnight at 4°C followed by extensive wash with IP buffer. Immunoprecipitated complexes on the beads were dissolved in 70 μl of 2 × SDS protein sample buffer containing 50 mM DTT. Alternatively, the immunoprecipitated protein complexes on the beads were eluted by incubation with 50 μl of 100 μg/ml 3×Flag (Sigma-Aldrich) in IP buffer for 2 h at 4°C. The eluant in the supernatant after spinning was collected and mixed with 20 μl of 5 x SDS protein buffer containing 50 mM DTT. All samples were heat-denatured at 95°C for 5–10 min before SDS-PAGE. Western blotting for individual proteins was carried out using the 3–5% input lysates and 30%-50% immunoprecipitated proteins. Densitometric quantification of the individual protein band intensity was performed using Image J software (NIH).

### IFA and confocal microscopy

Adherent HeLa and HEK293 cells were grown directly on glass coverslips. The non-adherent BCBL1 cells were immobilized by spotting of cells suspension on poly-D-lysine-treated glass coverslips. Immunofluorescence staining was performed as described previously [50,57,115]. Briefly, the cells were washed with PBS, fixed with 4% paraformaldehyde, permeabilized with 0.5% Triton X-100 and blocked with 2% BSA (bovine serum albumin, Promega, Madison, WI) dissolved in Tris-buffered saline containing 0.05% of Tween-20 (TTBS). Primary antibodies diluted in blocking buffer were incubated with slides overnight at 4°C. AlexaFluor-conjugated secondary antibodies (1: 500, ThermoFisher Scientific) were diluted in blocking solution and incubated with slides at 37°C in humidified chamber. The slides were washed with TTBS and before mounting the cells nuclei were visualized by 5 min counterstaining with wash buffer containing Hoechst dye 33342 (Sigma-Aldrich). Confocal fluorescence images were collected with a Zeiss LSM780 laser-scanning microscope (Carl Zeiss, Inc., Thornwood, NY) equipped with 20x Plan-Apochromat (numerical aperture, 0.8) and 63x Plan-Apochromat (numerical aperture, 1.4) objective lenses. The x-y pixel sizes of 0.4 and 0.07 μm and optical slice thicknesses of 1.5 and 0.9 μm were used to acquire confocal images with the 20× and 63× objectives lenses, respectively. Volume reconstructions were generated using the Imaris (version 8.0.2) image processing software (Bitplane, Inc., Concord, MA).

### Poly I:C labeling and pull-down assay

One microgram of reconstituted poly I:C was labelled using 10 units of T4 polynucleotide kinase (T4 PNK, Thermo Fisher Scientific) for 10 min at 37°C in a 25-μl reaction containing 25 μCi [γ-^32^P]-ATP (Perkin Elmer). The reaction was terminated by adding 5 mM EDTA to the reaction mixture and the labeled poly I:C was purified using an illustraMicrospin G-25 column (GE Healthcare, Marlborough, MA). In a pull-down assay, HeLa cells in a 10-cm culture dish were transfected with a Myc-Flag-tagged PKR expression vector or an empty vector. The Myc-Flag-tagged PKR protein in the HeLa cell lysate was immunoprecipitated using 80 μl of anti-mycEZview beads (Sigma-Aldrich) overnight at 4°C. The beads were then washed four times with IP buffer and subsequently resuspended in 100 μl of IP buffer. Approximately, 40 μl of the resuspended beads coated with PKR were incubated with a mixture of 100 ng [γ_-_^32^P]-labelled poly I:C and 500 ng of purified recombinant ORF57-Flag protein or BSA (negative control) in 750 μl of IP buffer for 2 h at room temperature. The beads were extensively washed with IP buffer, resuspended in 5 ml of liquid scintillation cocktail (CytoScint, MP Biochemicals, Santa Ana, CA) and radioactivity was counted by a liquid scintillation counter.

### In vitro PKR auto-phosphorylation assay

Recombinant full-length PKR was purified from HeLa cells in a 10-cm petri dish by by immunoprecipitation using 80 μl of anti-mycEZview beads as described above and subsequently dissolved in 100 μl of 1x kinase buffer (10 mM Tris-HCl [pH7.6], 50 mM KCl, 2 mM magnesium acetate, 20% glycerol). In a 32-μl kinase reaction, 12 μl of beads-attached PKR protein were first incubated with 200 ng of recombinant ORF57-Flag or BSA proteins for 10 min at room temperature. The resulting mixture was sequentially supplemented with 50 ng poly I:C, 1x kinase buffer supplemented with 0.83 mM MgCl_2_ and 20 μCi [γ_-_^32^P]-ATP. The reaction was incubated at 30°C for 10 min and terminated by addition of equal volume of 2 × SDS protein sample buffer. After SDS-PAGE the gel was mounted in an exposure cassette and analyzed by a PhosphorImager (GE Healthcare).

### In vitro phosphorylation of GST-eIF2α

HeLa cells with expression of Myc-Flag-tagged PKR in a 10-cm Petri dish were treated with arsenite to phosphorylate PKR. Myc-Flag-tagged PKR purified by immunoprecipitation as described above from the cells with (for activated PKR) or without (for inactive PKR) arsenite treatment was finally resuspended in 100 μl of 1x kinase buffer. Ten ul of the resuspended beads coated with PKR were mixed with 500 ng of recombinant ORF57 protein or BSA and incubated for 10 min at room temperature. The mixture was sequentially supplemented with 1x kinase buffer, 0.83 mM MgCl2, 50 ng GST-eIF2α (cat# H00001965-P01, Abnova), and 20 μCi [γ_-_^32^P]-ATP to a final volume of 32 μl. The kinase reaction involving eIF2α phosphorylation by phosphorylated (activated) PKR was allowed to proceed for 40 min at 30°C. The reaction was terminated by addition of 2x SDS sample buffer and the samples were resolved by SDS-PAGE. The gel was exposed to a PhosphorImager and an X-ray film for signal quantification.

### siRNA knockdown of PKR expression and induction of KSHV production in iSLK-BAC16 cells

KSHV infected iSLK-BAC16 cells [92] growing in a six-well plate (2.5 x 10^5^ cells/ well) were transfected twice, respectively, at an interval of 24 h with 40 nM of ON-TARGETplus SMART-pool PKR siRNAs targeting human PKR/EIF2AK2 (L-003527-00-0005, Dharmacon, GE Healthcare, Lafayette, CO) or ON-TARGETplus Non-targeting siRNA #1 negative control (D-001810-01, Dharmacon, GE Healthcare) using LipoJet transfection reagent (SL100468, SignaGen Laboratories, Gaithersburg, MD). Total cell extract was collected 24 h after the second siRNA transfection to measure the knockdown efficiency by immunoblotting.

For KSHV virus production and titration assays, iSLK-BAC16 cells in a six-well plate without or with PKR siRNA transfection at 24 h of the second round of siRNA transfections described above were induced for KSHV lytic infection with 1 mM sodium Butyrate and 1 ug/ml doxycycline in 2 ml of DMEM medium. On the third day, fresh 2 ml of DMEM medium containing the same amount of sodium butyrate and doxycycline were added to make the culture medium in total of 4 ml per well for another two more days. For virus production and titration, the iSLK-BAC16 culture supernatants were harvested on the 5^th^ day after induction, cleared by centrifugation at 2000 rpm for 10 min, and filtered through Sterile Millex 0.45 μM filter units (cat# SLHA033SS, Millipore, Billerica, MA). 400 μl of the supernatants were used to infect HEK293 (5 x 10^5^ cells/ well) in a six-well plate. KSHV-infected GFP^+^ HEK293 cells at 48 h after infection were observed by a fluorescent microscopy and analyzed by flow cytometry.

## Supporting information

S1 Fig Expression of ORF57, but not RTA, ORF45 and LANA, prevent SG formation during KSHV infection in BCBL-1 cells or Bac36 cells. (A) BCBL-1 cells with KSHV latent infection (- VA) or lytic infection reactivated by VA (+ VA) do not display SG. Showing in this panel are representative BCBL-1 cells stained for TIA-1 for SG and ORF57 for viral lytic infection. The nuclei were counterstained with Hoechst dye. Scale bar = 10 μm. (B-D) SG induction by arsenite in KSHV-infected BCBL-1 and Bac36 cells. KSHV lytic infection in BCBL-1 cells was induced by valproic acid (VA, 1 mM) (B and C) and in Bac36 cells harboring an ORF57-null KSHV genome (Δ57) was induced by sodium butyrate (Bu, 3 mM) (D). After 24 h induction, the cells were left untreated or treated with 0.5 mM arsenite for 30 min and followed by IFA staining for the SG-specific markers TIA-1 (red color) (B-D), PABPC1 (B) or G3BP1 (C) (white color) and viral protein ORF57 (green color) in BCBL-1 cells (B, C), or viral LANA or ORF45 protein (white color) in Bac36 Δ57 cells (D). The nuclei were counterstained with Hoechst dye. Bar = 10 μm. (E-F) Sensitivity of SG formation to cycloheximide. Bac36-Δ57 cells described in (D) treated with 3 mM of sodium butyrate (Bu) for 24 h (E) or transfected with an RTA-expression vector (F) without Bu treatment for 24 h were induced by 0.5 mM of sodium arsenite for 30 min and followed by 1 h treatment with cycloheximide (CHX, 10 μM) or vehicle medium (no CHX). Then, the cells were fixed and stained with an anti-TIA-1 antibody for the presence of SG (E-F) or anti-RTA for ectopically expressed RTA (F). The cell nuclei were counterstained with Hoechst dye. Bar = 10 μm.
(PDF)

S2 Fig KSHV ORF57 alone is sufficient to inhibit SG formation in HeLa cells, but does not affect the expression of major components for SG formation. (A) Transfection and expression of ORF57 in HeLa cells do not induce SG formation. HeLa cells transfected with an ORF57-Flag expressing vector (pVM7) or an empty vector (pCMV-Flag 5.1) for 24 h were stained for ORF57, SG-specific TIA-1 (red) and PABPC1 (green) by each corresponding antibody. The nuclei were counterstained with Hoechst stain. Bar = 10 μm. (B) HeLa cells transfected with an ORF57-Flag expressing vector (pVM7) or an empty vector (pFLAG-CMV-5.1) for 24 h were treated with 0.5 mM arsenite for 30 min to induce SG formation. The cells were then stained for ORF57 (green), SG-specific markers TIA-1 (red) and G3BP1 (white) by each corresponding antibody. The nuclei were counterstained with Hoechst stain. Bar = 10 μm. (C) HeLa cells transfected with a Flag empty vector (-) or an ORF57-Flag expressing (+) vector were treated with (+) or without (-) arsenite for 30 min before sample preparation. Expression of TIA-1, PABPC1, GAPDH and ORF57 in each sample was examined by Western blot analysis using each corresponding antibody. GAPDH served as a loading control. (D) ORF57 does not induce the cleavage or affect the expression of G3BP1. Cell lysates prepared from HeLa or HEK293 cells transfected with an empty vector (-) or an ORF57-Flag expressing (+) vector were blotted for the expression of G3BP1 and ORF57 using each corresponding antibody. β-actin served as a loading control. (E) ORF57 does not affect the expression and phosphorylation of eIF4E in HeLa cells. The cells were transfected as described above and blotted for the expression of total eIF4E and phosphorylated eIF4E using each corresponding antibody.
(TIF)

S3 Fig ORF57 inhibits TIA-1 insolubilization during stress. (A) Schematic flow of the steps followed to separate soluble and insoluble TIA-1 after arsenite exposure of HeLa cells. (B) ORF57, but not its mutant, prevents TIA-1 insolubilization. HeLa cells transfected with a Flag empty vector (-) or a Flag-tagged ORF57- or ORF57 mt-expressing vector were treated with (+) or without (-) arsenite for 30 min before sample preparation. The lysed cell samples were centrifuged at 15800 x g for 15 min to separate the supernatants (S) from insoluble pellets (P) of the same cell lysate. The fractionated S and P in SDS sample buffer were resolved by SDS-PAGE and blotted for the relative level of Flag-ORF57 and TIA-1 (lower panel). Tubulin served as a loading control. (C) Kinetic insolubilization of TIA-1 in HeLa cells induced by arsenite and prevention of the TIA-1 insolubilization by ORF57. HeLa cells with or without ORF57 expression were induced by arsenite for 0, 5, 10, 20 or 30 min for SG formation. Cell lysates from each time point were prepared and separated as soluble and insoluble fractions as described in (B). ORF57 in total cell lysate and ORF57 and TIA-1 in the insoluble pellets were blotted. Tubulin served as a loading control.
(TIF)

S4 Fig Inhibition of arsenite-induced PKR phosphorylation and SG formation by a PKR inhibitor. (A) Inhibition of PKR phosphorylation by a PKR inhibitor. HeLa cells were treated with medium containing different doses (1, 10, or 100 μM) of a PKR inhibitor (PKR_i_) or inhibitor control (PKR_c_) or 0.2% DMSO for 2 h and then, after washing once with PBS, treated with 0.5 mM arsenite for 30 min. The cells were rinsed with PBS again, directly lysed in 2 × SDS sample buffer, and blotted for p-PKR and total PKR. Actin served as a sample loading control. (B-C) Inhibition of SG formation by a PKR inhibitor. HeLa cells treated with 100 μM of PKR_i_, PKR_c_, or 0.2% DMSO for 1h were treated with 0.5 mM of arsenite for 30 min and then stained for TIA-1-specific SG. The nuclei were counterstained with Hoechst. Images were captured using confocal microscopy (B). Scale bar = 10 μm. 100 cells with SG were counted and averaged for number of SG per cell in each experimental group (C). The mean ± SD in the bar graph are derived from three independent replicates (C). (D) Arsenite-induced SG formation in BCBL-1 cells is PKR-dependent. BCBL-1 cells with latent KSHV infection and pretreated with 100 μM of PKR inhibitor (PKR_i_), inhibitor control (PKR_c_), or 0.2% DMSO (vehicle) for 1 h were induced for SG formation by 0.5 mM of arsenite for 30 min and then stained for SG using anti-TIA-1 antibody. BCBL-1 cells with lytic KSHV induction by VA for the expression of ORF57 were also treated with arsenite to serve as a comparative control to PKRi inhibition of SG formation. The nuclei were counterstained with Hoechst. Images were captured using confocal microscopy. The scale bar = 10μm.
(PDF)

S5 Fig ORF57 inhibits phosphorylation of eIF2α, PKR and TLR3. (A) Poly I:C dose-dependent phosphorylation of eIF2α. HeLa cells transfected with an increasing amount of poly I:C for 8 h were blotted for p-eIF2α and eIF2α. GAPDH served as a loading control. (B) Arsenite-induced oxidative stress does not induce activation and phosphorylation of PERK. HeLa cells transfected with an empty (-) or ORF57 expressing (ORF57) vector were treated with arsenite. The cell lysates were examined by Western blot analysis for PERK, p-PERK, ORF57 and GAPDH. GAPDH served as sample loading control. (C) ORF57 inhibits the arsenite- and poly I:C induced phosphorylation of PKR in HEK293 cells. HEK293 cells with or without ORF57 expression for 24 h were treated with arsenite or transfected with poly I:C and blotted for p-PKR, PKR, and ORF57. β-actin served as a loading control. (D) Kinetic profile of arsenite-induced phosphorylation of PKR and eIF2α. HeLa cells transfected with an empty or ORF57 expression vector. Twenty-four hours later, cells were subjected to arsenite treatment for 0, 15 or 30 mins. Cells lysates were analyzed by Western blotting to detect levels of p-PKR, PKR, p-eIF2α and eIF2α. GAPDH served as a loading control. (E) ORF57 inhibits the poly I:C-induced phosphorylation of both PKR and TLR3. HeLa cells with or without ORF57 expression for 20 h were transfected with poly I:C (1 μg) for 8 h and blotted for p-PKR, p-TLR3, and ORF57. GAPDH served as a loading control.
(PDF)

S6 Fig Inhibition of PKR-mediated, but not heat-mediated SG formation by ORF57. HeLa cells transfected by an ORF57 expression vector (pVM7) for 24 h were treated with arsenite (0.5 mM) for 30 min, poly I:C (1 μg) for 8 h or heat at 44°C for 40 min to induce SG formation. The cells were stained for ORF57 (green), TIA-1 (red) by each corresponding antibody. A representative imaging field of cells from each induction condition is shown. Two magnified images on individual cells with or without ORF57 expression are shown in the right panels. The nuclei were counterstained with Hoechst dye. Scale bar = 10 μm.
(PDF)

S1 Video KSHV ORF57 inhibits arsenite-induced SG formation in HeLa cells. HeLa cells transfected with an ORF57 expression vector for 24 h were treated with arsenite, fixed with paraformaldehyde, processed for IFA against SG-specific TIA-1 (red) using confocal microscopy, and post-processed using LSM image browser software. Showing in the video are confocal cell images in rotation with ORF57 fluorescence in green and nuclei (Hoechst dye) in blue.
(MP4)

S2 Video KSHV ORF57 inhibits arsenite-induced SG formation in HeLa cells. HeLa cells transfected with an ORF57 expression vector for 24 h were treated with arsenite and fixed with paraformaldehyde and then processed for IFA against TIA-1 (red) and serial z-stack images were taken at 0.50 μm intervals using confocal microscopy and post-processed using LSM image browser software. ORF57 fluorescence is shown in green and nuclei (Hoechst dye) are shown in blue.
(MP4)
