# Supplementary material for: KSHV inhibits stress granule formation by viral ORF57 blocking PKR activation
Source: PLoS Pathog. 2017 Oct 30;13(10):e1006677. doi: 10.1371/journal.ppat.1006677 (PMC5679657; doi:10.1371/journal.ppat.1006677)
Supplement: S1 Fig — (A) BCBL-1 cells with KSHV latent infection (- VA) or lytic infection reactivated by VA (+ VA) do not display SG. Showing in this panel are representative BCBL-1 cells stained for TIA-1 for SG and ORF57 for viral lytic infection. The nuclei were counterstained with Hoechst dye. Scale bar = 10 μm. (B-D) SG induction by arsenite in KSHV-infected BCBL-1 and Bac36 cells. KSHV lytic infection in BCBL-1 cells was induced by valproic acid (VA, 1 mM) (B and C) and in Bac36 cells harboring an ORF57-null KSHV genome (Δ57) was induced by sodium butyrate (Bu, 3 mM) (D). After 24 h induction, the cells were left untreated or treated with 0.5 mM arsenite for 30 min and followed by IFA staining for the SG-specific markers TIA-1 (red color) (B-D), PABPC1 (B) or G3BP1 (C) (white color) and viral protein ORF57 (green color) in BCBL-1 cells (B, C), or viral LANA or ORF45 protein (white color) in Bac36 Δ57 cells (D). The nuclei were counterstained with Hoechst dye. Bar = 10 μm. (E-F) Sensitivity of SG formation to cycloheximide. Bac36-Δ57 cells described in (D) treated with 3 mM of sodium butyrate (Bu) for 24 h (E) or transfected with an RTA-expression vector (F) without Bu treatment for 24 h were induced by 0.5 mM of sodium arsenite for 30 min and followed by 1 h treatment with cycloheximide (CHX, 10 μM) or vehicle medium (no CHX). Then, the cells were fixed and stained with an anti-TIA-1 antibody for the presence of SG (E-F) or anti-RTA for ectopically expressed RTA (F). The cell nuclei were counterstained with Hoechst dye. Bar = 10 μm. (PDF) [file ppat.1006677.s001.pdf]

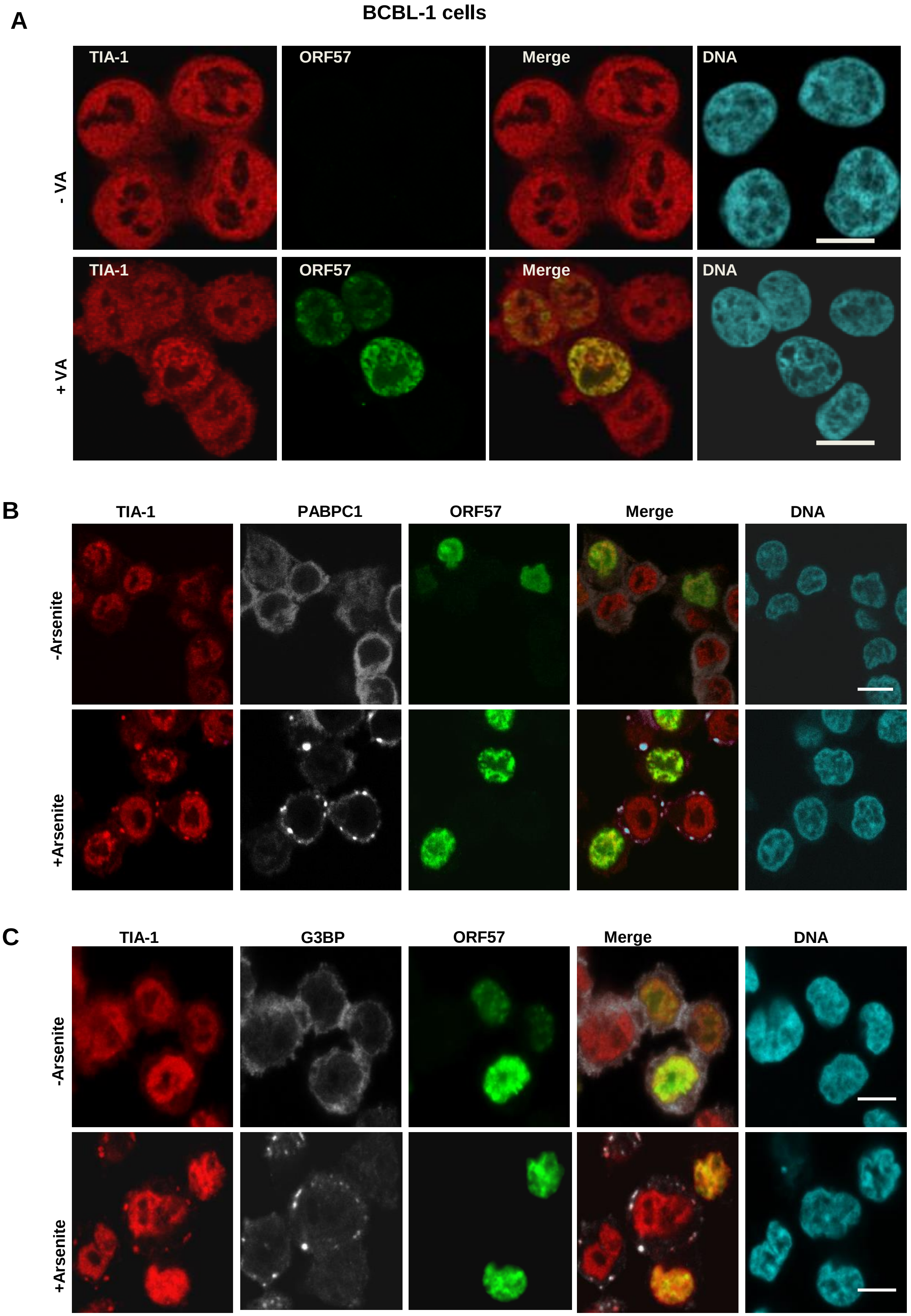
A
BCBL-1 cells
TIA-1
ORF57
Merge
DNA
- VA
+ VA
B
TIA-1
PABPC1
ORF57
Merge
DNA
-Arsenite
+Arsenite
C
TIA-1
G3BP
ORF57
Merge
DNA
-Arsenite
+Arsenite

S1 Fig.

D BAC36-Δ57–3 mM Bu for 24 h/0.5 mM arsenite for 30 min

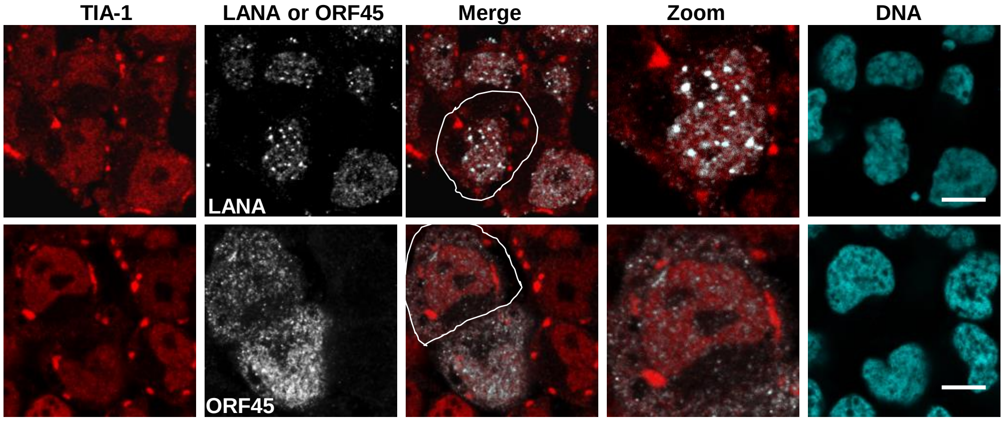

E Bac36-Δ57–3 mM Bu for 24 h/0.5 mM arsenite for 30 min

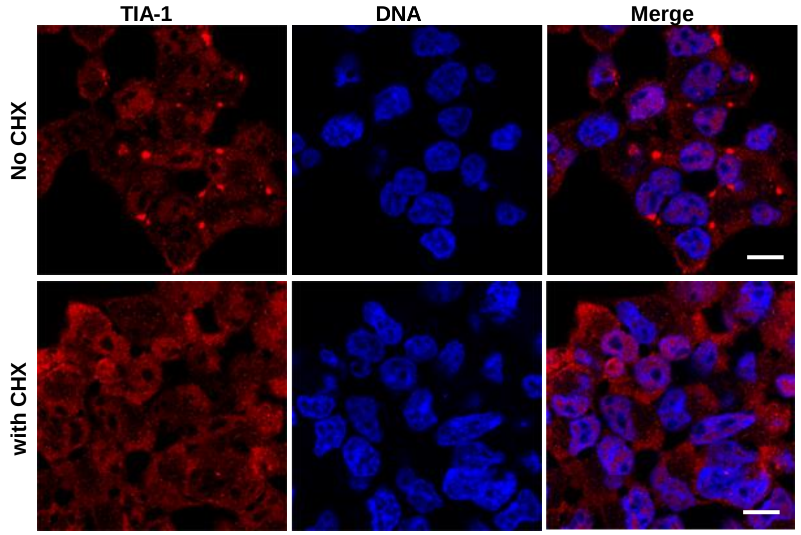

F Bac36-Δ57 – RTA expression only, no Bu and no arsenite

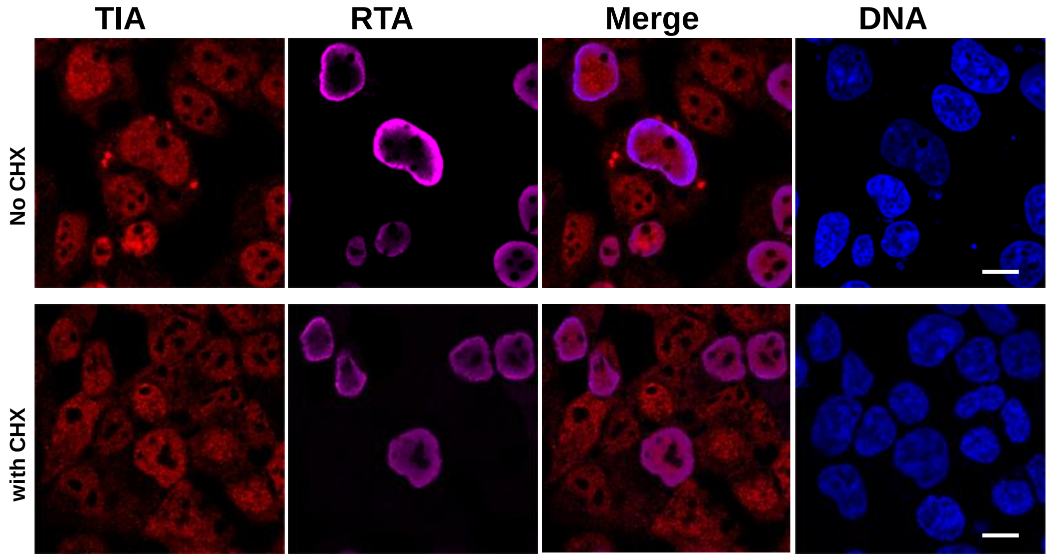

S1 Fig.
