# Supplementary figures and images for: KSHV inhibits stress granule formation by viral ORF57 blocking PKR activation
Source: PLoS Pathog. 2017 Oct 30;13(10):e1006677. doi: 10.1371/journal.ppat.1006677 (PMC5679657; doi:10.1371/journal.ppat.1006677)

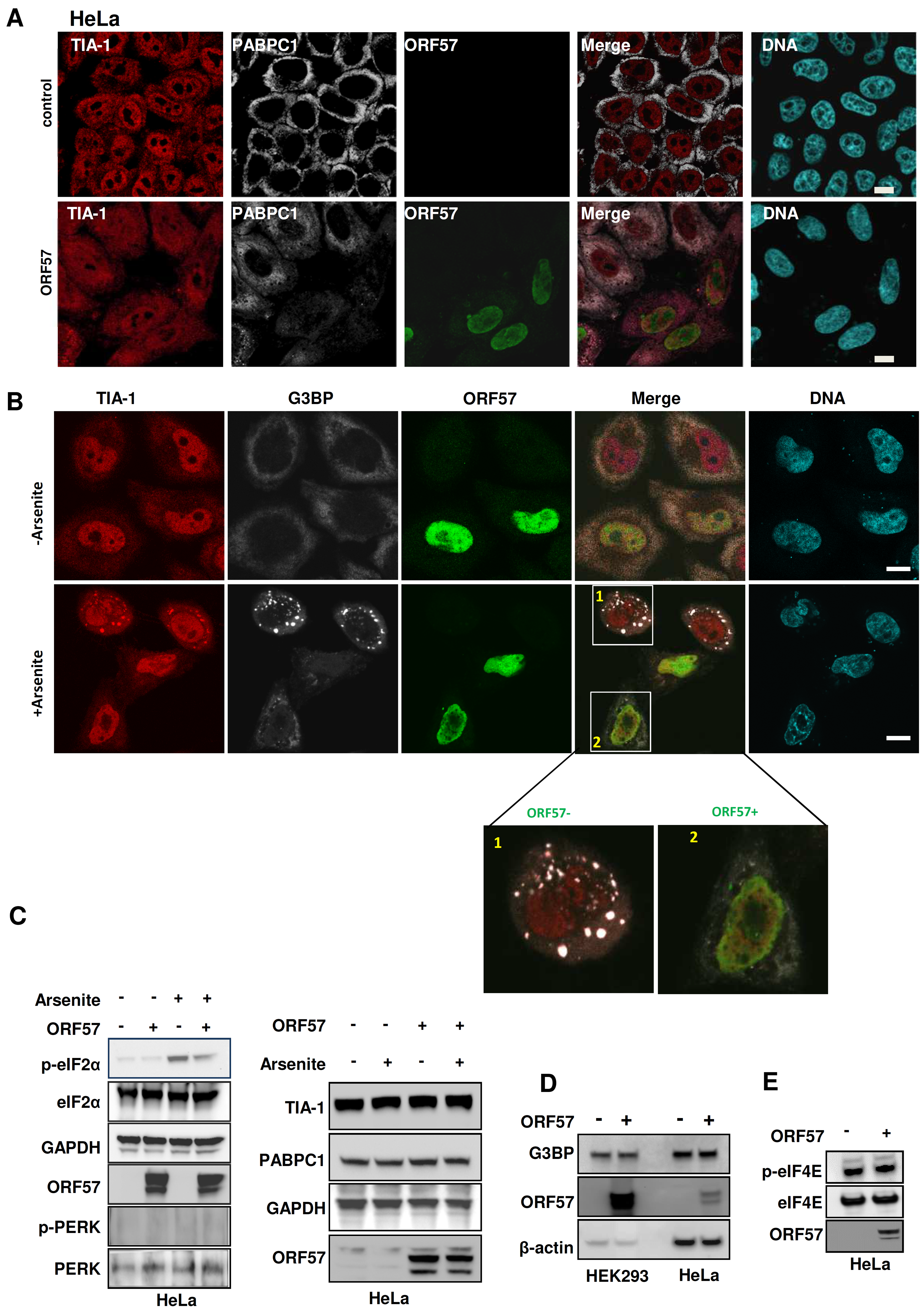

Supplement: S2 Fig — (A) Transfection and expression of ORF57 in HeLa cells do not induce SG formation. HeLa cells transfected with an ORF57-Flag expressing vector (pVM7) or an empty vector (pCMV-Flag 5.1) for 24 h were stained for ORF57, SG-specific TIA-1 (red) and PABPC1 (green) by each corresponding antibody. The nuclei were counterstained with Hoechst stain. Bar = 10 μm. (B) HeLa cells transfected with an ORF57-Flag expressing vector (pVM7) or an empty vector (pFLAG-CMV-5.1) for 24 h were treated with 0.5 mM arsenite for 30 min to induce SG formation. The cells were then stained for ORF57 (green), SG-specific markers TIA-1 (red) and G3BP1 (white) by each corresponding antibody. The nuclei were counterstained with Hoechst stain. Bar = 10 μm. (C) HeLa cells transfected with a Flag empty vector (-) or an ORF57-Flag expressing (+) vector were treated with (+) or without (-) arsenite for 30 min before sample preparation. Expression of TIA-1, PABPC1, GAPDH and ORF57 in each sample was examined by Western blot analysis using each corresponding antibody. GAPDH served as a loading control. (D) ORF57 does not induce the cleavage or affect the expression of G3BP1. Cell lysates prepared from HeLa or HEK293 cells transfected with an empty vector (-) or an ORF57-Flag expressing (+) vector were blotted for the expression of G3BP1 and ORF57 using each corresponding antibody. β-actin served as a loading control. (E) ORF57 does not affect the expression and phosphorylation of eIF4E in HeLa cells. The cells were transfected as described above and blotted for the expression of total eIF4E and phosphorylated eIF4E using each corresponding antibody. (TIF) [file ppat.1006677.s002.tif]

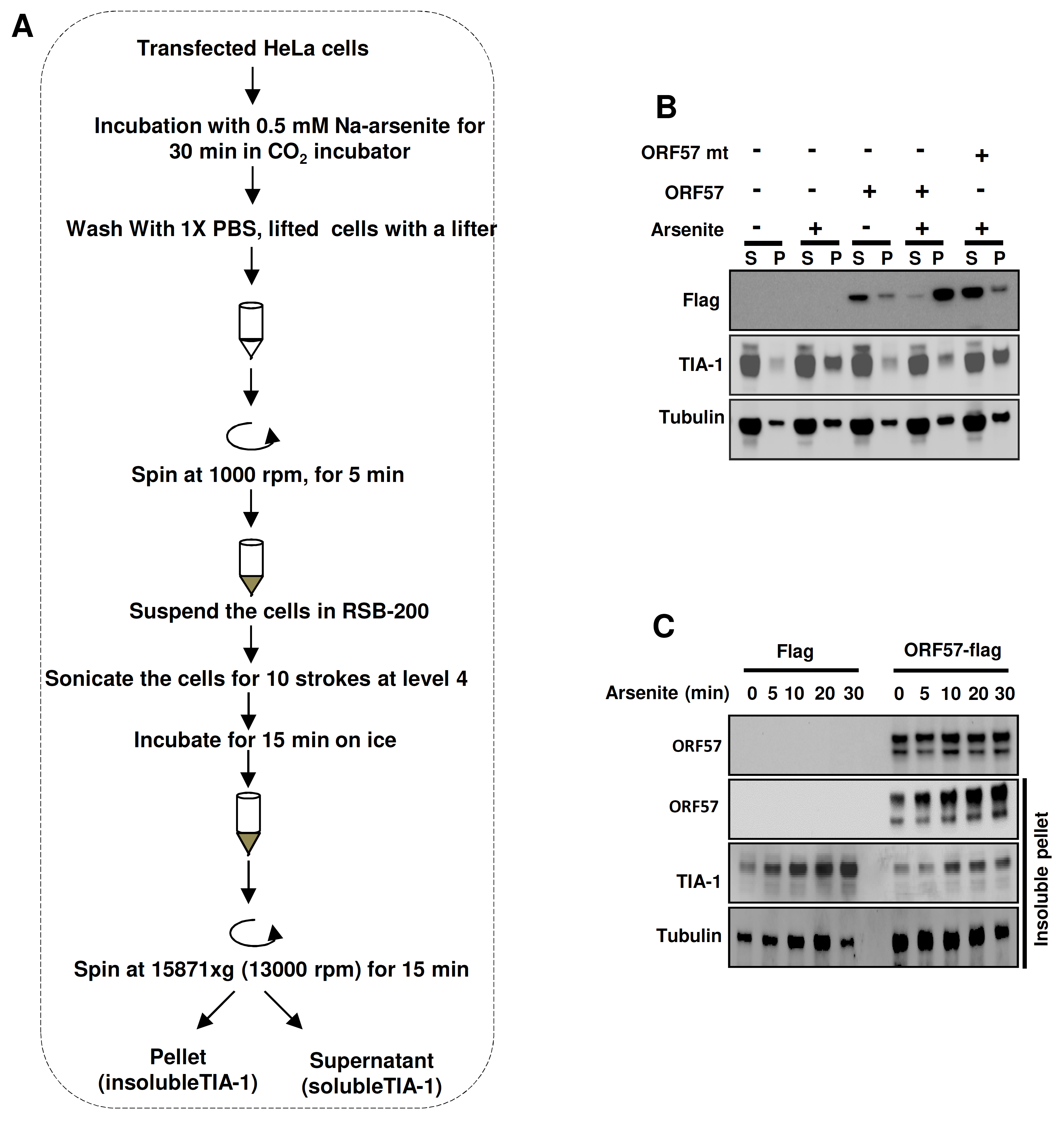

Supplement: S3 Fig — (A) Schematic flow of the steps followed to separate soluble and insoluble TIA-1 after arsenite exposure of HeLa cells. (B) ORF57, but not its mutant, prevents TIA-1 insolubilization. HeLa cells transfected with a Flag empty vector (-) or a Flag-tagged ORF57- or ORF57 mt-expressing vector were treated with (+) or without (-) arsenite for 30 min before sample preparation. The lysed cell samples were centrifuged at 15800 x g for 15 min to separate the supernatants (S) from insoluble pellets (P) of the same cell lysate. The fractionated S and P in SDS sample buffer were resolved by SDS-PAGE and blotted for the relative level of Flag-ORF57 and TIA-1 (lower panel). Tubulin served as a loading control. (C) Kinetic insolubilization of TIA-1 in HeLa cells induced by arsenite and prevention of the TIA-1 insolubilization by ORF57. HeLa cells with or without ORF57 expression were induced by arsenite for 0, 5, 10, 20 or 30 min for SG formation. Cell lysates from each time point were prepared and separated as soluble and insoluble fractions as described in (B). ORF57 in total cell lysate and ORF57 and TIA-1 in the insoluble pellets were blotted. Tubulin served as a loading control. (TIF) [file ppat.1006677.s003.tif]

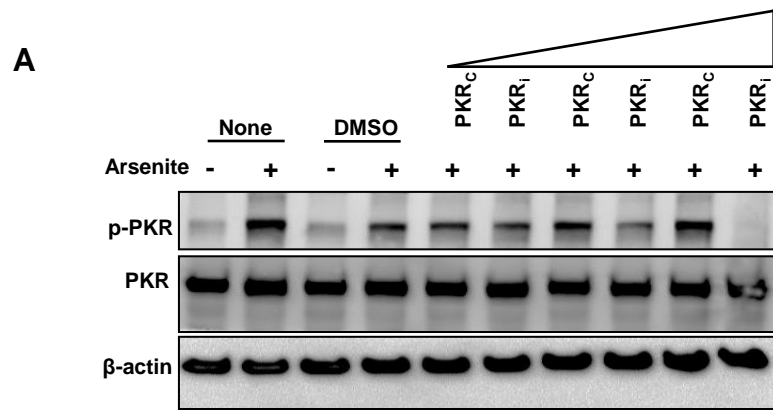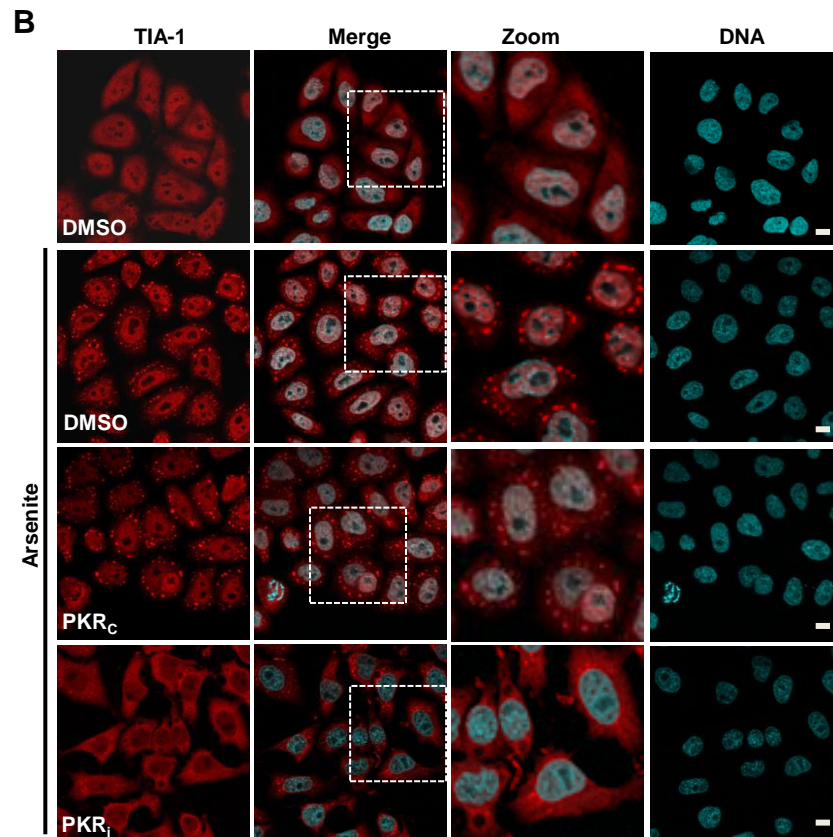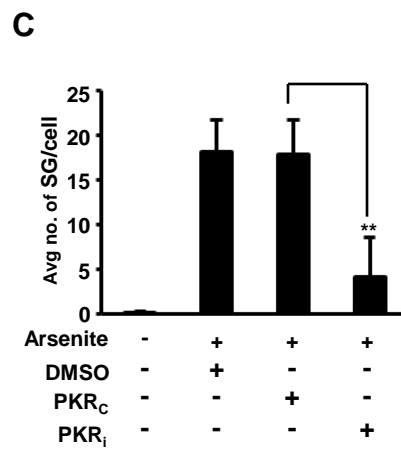

**S4 Fig.**

D

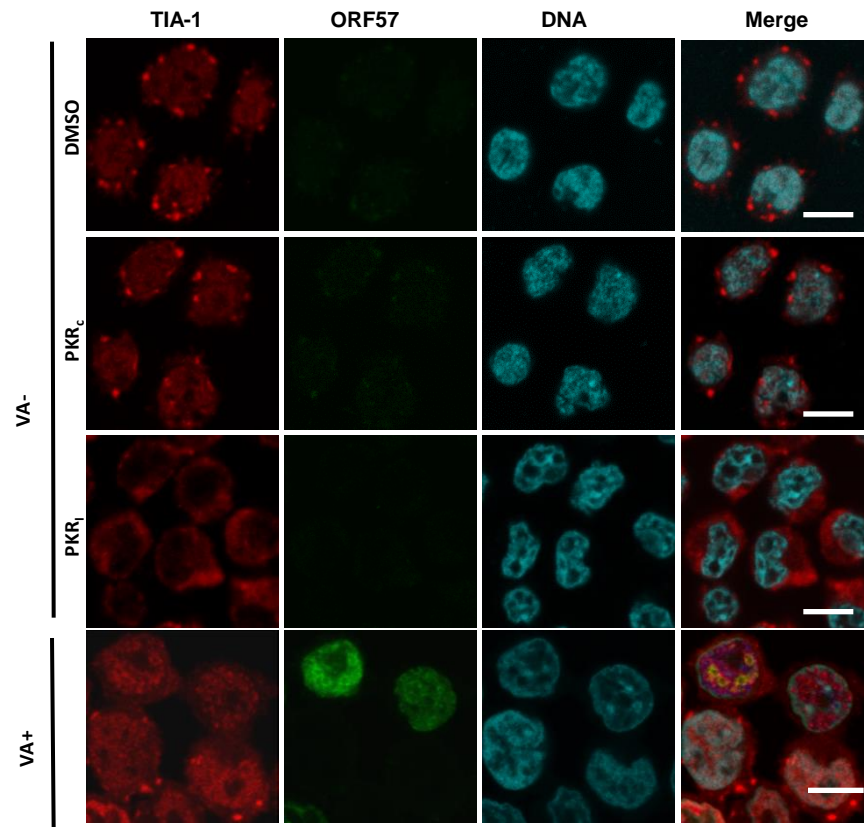

S4 Fig.

Supplement: S4 Fig — (A) Inhibition of PKR phosphorylation by a PKR inhibitor. HeLa cells were treated with medium containing different doses (1, 10, or 100 μM) of a PKR inhibitor (PKRi) or inhibitor control (PKRc) or 0.2% DMSO for 2 h and then, after washing once with PBS, treated with 0.5 mM arsenite for 30 min. The cells were rinsed with PBS again, directly lysed in 2 × SDS sample buffer, and blotted for p-PKR and total PKR. Actin served as a sample loading control. (B-C) Inhibition of SG formation by a PKR inhibitor. HeLa cells treated with 100 μM of PKRi, PKRc, or 0.2% DMSO for 1h were treated with 0.5 mM of arsenite for 30 min and then stained for TIA-1-specific SG. The nuclei were counterstained with Hoechst. Images were captured using confocal microscopy (B). Scale bar = 10 μm. 100 cells with SG were counted and averaged for number of SG per cell in each experimental group (C). The mean ± SD in the bar graph are derived from three independent replicates (C). (D) Arsenite-induced SG formation in BCBL-1 cells is PKR-dependent. BCBL-1 cells with latent KSHV infection and pretreated with 100 μM of PKR inhibitor (PKRi), inhibitor control (PKRc), or 0.2% DMSO (vehicle) for 1 h were induced for SG formation by 0.5 mM of arsenite for 30 min and then stained for SG using anti-TIA-1 antibody. BCBL-1 cells with lytic KSHV induction by VA for the expression of ORF57 were also treated with arsenite to serve as a comparative control to PKRi inhibition of SG formation. The nuclei were counterstained with Hoechst. Images were captured using confocal microscopy. The scale bar = 10μm. (PDF) [file ppat.1006677.s004.pdf]

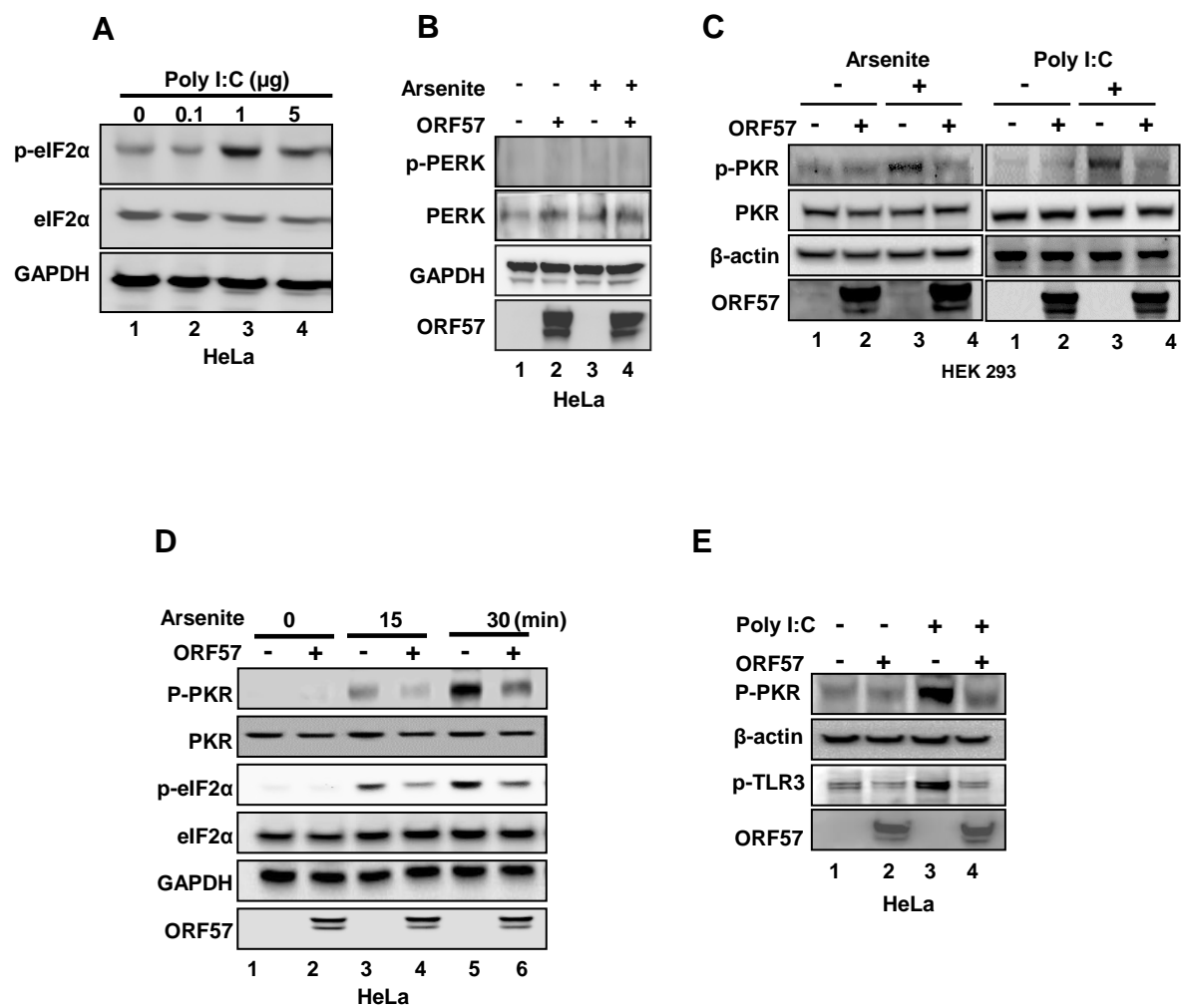

**S5 Fig.**

Supplement: S5 Fig — (A) Poly I:C dose-dependent phosphorylation of eIF2α. HeLa cells transfected with an increasing amount of poly I:C for 8 h were blotted for p-eIF2α and eIF2α. GAPDH served as a loading control. (B) Arsenite-induced oxidative stress does not induce activation and phosphorylation of PERK. HeLa cells transfected with an empty (-) or ORF57 expressing (ORF57) vector were treated with arsenite. The cell lysates were examined by Western blot analysis for PERK, p-PERK, ORF57 and GAPDH. GAPDH served as sample loading control. (C) ORF57 inhibits the arsenite- and poly I:C induced phosphorylation of PKR in HEK293 cells. HEK293 cells with or without ORF57 expression for 24 h were treated with arsenite or transfected with poly I:C and blotted for p-PKR, PKR, and ORF57. β-actin served as a loading control. (D) Kinetic profile of arsenite-induced phosphorylation of PKR and eIF2α. HeLa cells transfected with an empty or ORF57 expression vector. Twenty-four hours later, cells were subjected to arsenite treatment for 0, 15 or 30 mins. Cells lysates were analyzed by Western blotting to detect levels of p-PKR, PKR, p-eIF2α and eIF2α. GAPDH served as a loading control. (E) ORF57 inhibits the poly I:C-induced phosphorylation of both PKR and TLR3. HeLa cells with or without ORF57 expression for 20 h were transfected with poly I:C (1 μg) for 8 h and blotted for p-PKR, p-TLR3, and ORF57. GAPDH served as a loading control. (PDF) [file ppat.1006677.s005.pdf]

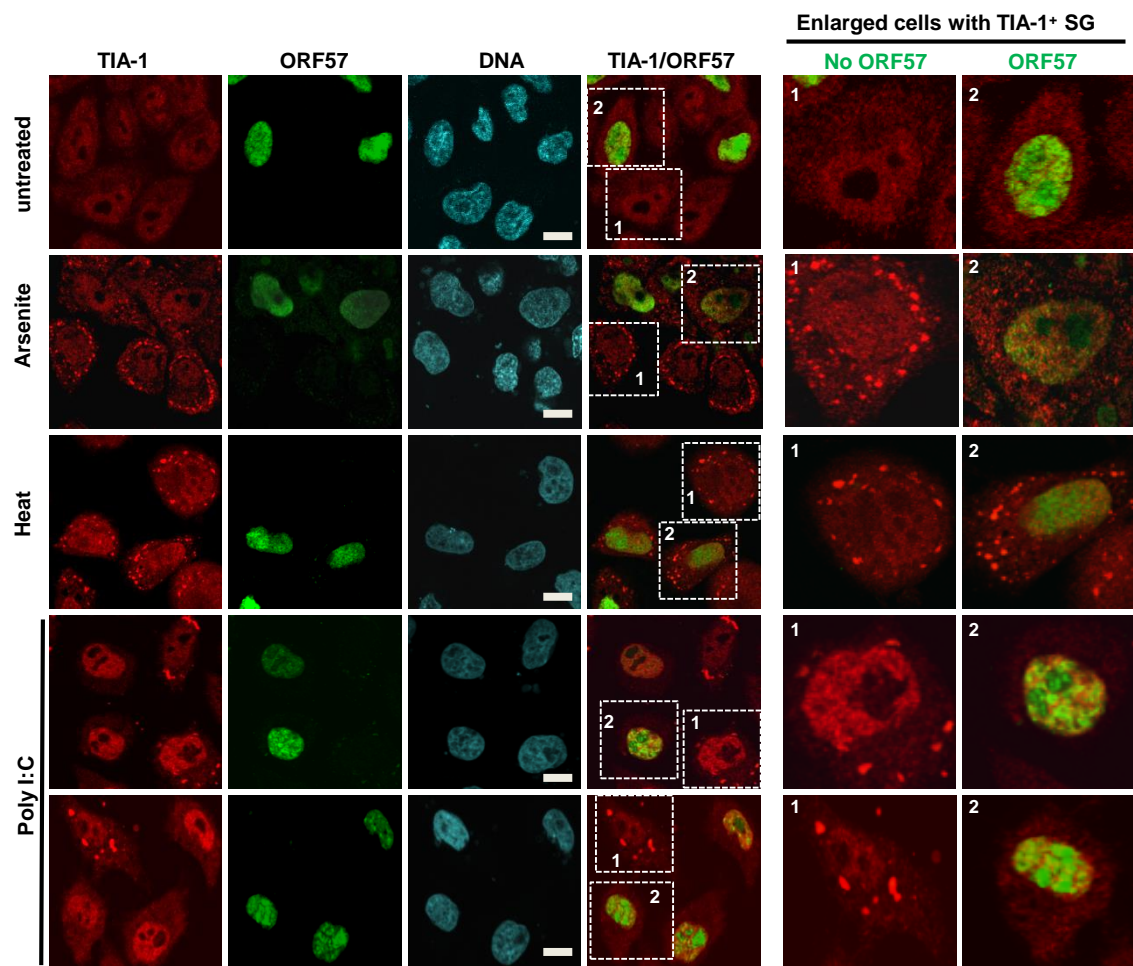

S6 Fig.

Supplement: S6 Fig — HeLa cells transfected by an ORF57 expression vector (pVM7) for 24 h were treated with arsenite (0.5 mM) for 30 min, poly I:C (1 μg) for 8 h or heat at 44°C for 40 min to induce SG formation. The cells were stained for ORF57 (green), TIA-1 (red) by each corresponding antibody. A representative imaging field of cells from each induction condition is shown. Two magnified images on individual cells with or without ORF57 expression are shown in the right panels. The nuclei were counterstained with Hoechst dye. Scale bar = 10 μm. (PDF) [file ppat.1006677.s006.pdf]
